# A comprehensive analysis of translational misdecoding pattern and its implication on genetic code evolution

**DOI:** 10.1093/nar/gkad707

**Published:** 2023-08-28

**Authors:** Takayuki Katoh, Hiroaki Suga

**Affiliations:** Department of Chemistry, Graduate School of Science, The University of Tokyo, 7-3-1 Hongo, Bunkyo-ku, Tokyo 113-0033, Japan; Department of Chemistry, Graduate School of Science, The University of Tokyo, 7-3-1 Hongo, Bunkyo-ku, Tokyo 113-0033, Japan

## Abstract

The universal genetic code is comprised of 61 sense codons, which are assigned to 20 canonical amino acids. However, the evolutionary basis for the highly conserved mapping between amino acids and their codons remains incompletely understood. A possible selective pressure of evolution would be minimization of deleterious effects caused by misdecoding. Here we comprehensively analyzed the misdecoding pattern of 61 codons against 19 noncognate amino acids where an arbitrary amino acid was omitted, and revealed the following two rules. (i) If the second codon base is U or C, misdecoding is frequently induced by mismatches at the first and/or third base, where any mismatches are widely tolerated; whereas misdecoding with the second-base mismatch is promoted by only U-G or C-A pair formation. (ii) If the second codon base is A or G, misdecoding is promoted by only G-U or U-G pair formation at the first or second position. In addition, evaluation of functional/structural diversities of amino acids revealed that less diverse amino acid sets are assigned at codons that induce more frequent misdecoding, and vice versa, so as to minimize deleterious effects of misdecoding in the modern genetic code.

## Graphical Abstract

**Graphical Abstract:**
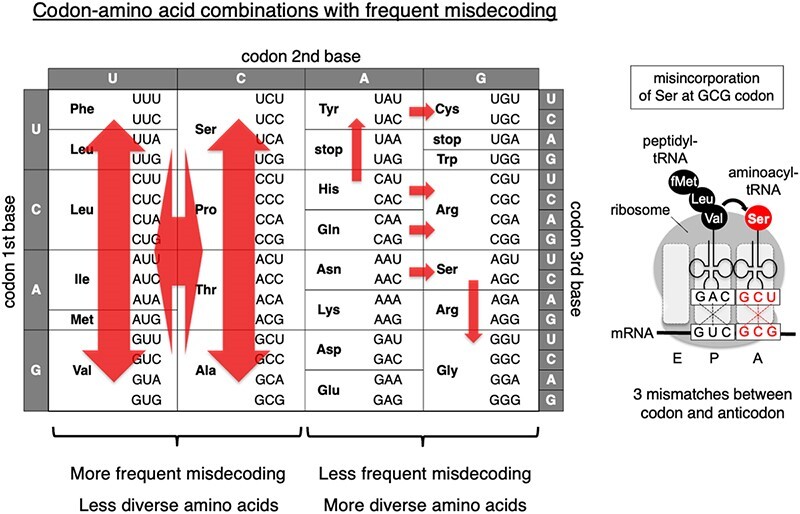

## INTRODUCTION

The genetic code defines the relationship between codons comprised of a triplet of four nucleotides and the corresponding amino acids. In the modern genetic code, which is highly conserved among the three domains of life, 20 canonical amino acids are assigned to 61 sense codons (Supplementary Figure S1, bottom). This assignment is a consequence of evolution by natural selection. However, there is an argument about what kind of selective pressure has been applied to the evolution. It is generally believed that the primordial genetic code had started with a smaller set of amino acids than the current 20. According to Miller-Urey-type prebiotic synthesis experiments and analyses of meteorite samples, amino acids with thermodynamically more stable structures, such as Ala, Asp, Glu, Gly, Ile, Leu, Pro, Ser, Thr and Val, likely emerged earlier on the Earth than the rest of the 20 amino acids (1–3). In the 2-1-3 model and the four-column theory, the four simplest amino acids, Val, Ala, Asp and Gly, were first assigned to the code comprised of four columns, NUN, NCN, NAN and NGN, respectively, utilizing only the second base to distinguish each other (Supplementary Figure S1, 4 column code) (4,5). Then, the amino acids that emerged later, such as Leu, Ile, Ser, Pro, and Thr, were added to the code utilizing the first base for discrimination (Supplementary Figure S1, 16 supercodon code). Eventually, the rest of the 20 amino acids were added to the code, where the codon table was divided into 64 codons using the third base (Supplementary Figure S1, 64 codon code).

In the modern genetic code, codons bearing U at the second position (= NUN column) are all assigned to functionally similar amino acids, i.e. hydrophobic amino acids: Phe, Leu, Ile, Met and Val. Codons with C at the second position (= NCN column) are assigned to amino acids with small sidechains: Ser, Pro, Thr and Ala. This array of assignment strengthens the robustness of NUN and NCN columns to decoding errors at the first and third bases; even if misdecoding occurs in a vertical direction in the codon table, deleterious effects caused by amino acid substitution would be minimal owing to the functional and structural similarities of amino acids assigned within the same column. Thus, it has been suggested that the genetic code has evolved so as to minimize the effect of decoding error, which is referred to as the error minimization theory (6–8). However, amino acids assigned at NAN and NGN columns are functionally and structurally more diverse than those at NUN and NCN, and therefore the robustness of NAN and NGN would not be high. To date, researchers in this field have estimated robustness of the modern genetic code to misdecoding using various calculation strategies (9–11). Such studies showed that a huge number of alternative assignments are possible with even higher robustness than the modern genetic code, i.e. the current array of amino acid assignment has not been fully optimized, arguing against the validity of error minimization theory (12–14).

An issue for these robustness estimation studies resides in insufficient consideration of the misdecoding pattern, which may lead to underestimation of the robustness of the modern genetic code. Codon-amino acid combinations with higher misdecoding frequencies would exert more deleterious effects on the code, whereas those with lower frequencies would have smaller effects. For instance, comparison of misdecoding patterns within NYN and NRN columns would explain the difference in functional/structural diversities of amino acids assigned to these columns. Position and number of mismatches between codon and anticodon that cause misdecoding should also be monitored as errors in both the horizontal and diagonal directions can also occur. If such horizontal/diagonal errors are also frequently induced, robustness of the code to error may decrease. Since there are 61 sense codons and 19 noncognate amino acids for each codon, 1159 codon-amino acid combinations could be considered for evaluation of misdecoding frequency. However, previous studies have monitored misdecoding at a limited number of selected codons, which were not sufficient to establish solid rules for the misdecoding pattern (15–17). We therefore set out to conduct a comprehensive analysis of the misdecoding susceptibility of all 61 sense codons to each of the 19 noncognate amino acids. Here we systematically evaluate all 1159 misdecoding codon-amino acid combinations and establish encompassing rules that clearly describe the misdecoding pattern. In the context of the elucidated pattern and the diversity of amino acids assigned to the code, we then assess the robustness of the modern genetic code to misdecoding.

## MATERIALS AND METHODS

### Preparation of template DNA for mRNA transcription, tRNA and eFx

Template DNAs for transcription of mRNA, tRNA and a flexizyme variant called eFx were prepared by extension and PCR using *Taq* DNA polymerase (18). Reverse PCR primers for tRNA were modified with 2′-*O*-methylation at the second nucleotide from the 5′-end (See Supplementary Table S1 for the primer sequences). The PCR products were purified by phenol/chloroform extraction and ethanol precipitation. Transcription of tRNA and eFx was carried out at 37°C for overnight in a 2-ml reaction mixture of the following composition: 40 mM Tris–HCl (pH 8.0), 22.5 mM MgCl_2_, 1 mM dithiothreitol (DTT), 1 mM spermidine, 0.01% Triton X-100, 3.75 or 5 mM nucleoside triphosphate (NTP) mix, 5 or 0 mM guanosine monophosphate (GMP), 0.04 U/μl RNasin RNase inhibitor (Promega), 0.12 μM T7 RNA polymerase, and 2-ml-scale PCR product. Concentration of NTP mix was 3.75 mM or 5 mM and that of GMP was 5 or 0 mM for tRNA and eFx, respectively. The reaction mix was treated with RQ1 DNase (Promega) at 37°C for 30 min and purified by denaturing polyacrylamide gel electrophoresis.

### Preparation of aminoacyl-tRNA

*N*-Acetylphenylalanine-cyanomethyl ester (AcPhe-CME) and 4-iodophenylalanine-cyanomethyl ester (Phe^I^-CME) were prepared by previously reported methods (19,20). eFx catalyzes aminoacylation of CME-activated amino acids onto arbitrary tRNAs. Aminoacylation of tRNA was carried out at 0°C for 2 h in 50 mM HEPES–KOH (pH 7.5), 600 mM MgCl_2_, 20% DMSO, 25 μM eFx, 25 μM tRNA and 5 mM AcPhe-CME or Phe^I^-CME. Aminoacyl-tRNA was recovered by ethanol precipitation, washed twice with 70% ethanol containing 0.1 M sodium acetate (pH 5.2), and dissolved in 1 mM sodium acetate (pH 5.2).

### *In vitro* translation of peptides

Translation of peptide was carried out at 37°C for 30 min in a 5 μl-scale flexible *in vitro* translation (FIT) system (21) of the following composition: 50 mM HEPES–KOH (pH 7.6), 100 mM potassium acetate, 12.6 mM magnesium acetate, 0.1 mM 10-formyl-5,6,7,8-tetrahydrofolic acid, 2 mM ATP, 2 mM GTP, 1 mM CTP, 1 mM UTP, 20 mM creatine phosphate, 2 mM spermidine, 1 mM DTT, 1.5 mg/ml *Escherichia coli* total tRNA, 1.2 μM *E. coli* ribosome, 0.6 μM methionyl-tRNA formyltransferase, 2.7 μM IF1, 0.4 μM IF2, 1.5 μM IF3, 0.26 μM EF-G, 10 μM EF-Tu/Ts, 0.25 μM RF2, 0.17 μM RF3, 0.5 μM RRF, 4 μg/ml creatine kinase, 3 μg/ml myokinase, 0.1 μM inorganic pyrophosphatase, 0.1 μM nucleotide diphosphate kinase, 0.1 μM T7 RNA polymerase, 0.73 μM AlaRS, 0.03 μM ArgRS, 0.38 μM AsnRS, 0.13 μM AspRS, 0.02 μM CysRS, 0.06 μM GlnRS, 0.23 μM GluRS, 0.09 μM GlyRS, 0.02 μM HisRS, 0.40 μM IleRS, 0.04 μM LeuRS, 0.11 μM LysRS, 0.03 μM MetRS, 0.68 μM PheRS, 0.16 μM ProRS, 0.04 μM SerRS, 0.09 μM ThrRS, 0.03 μM TrpRS, 0.02 μM TyrRS, 0.02 μM ValRS, 0.5 mM each amino acid mix and 1.6 μM DNA template. To prepare a vacant codon for misincorporation, the corresponding amino acid was removed from the above reaction mix. For translation of authentic peptides, all of the 20 amino acids were added to synthesize peptides of correct sequences. For isotope labeling, 0.5 mM [^13^C_6_]-Ile or [D_10_]-Leu was added in place of unlabeled Ile or Leu. In translation of mR2, Met and 10-formyl-5,6,7,8-tetrahydrofolic acid were removed and 25 μM AcPhe-tRNA^fMet^ was added. In translation of P1-Phe^I^ using mR1-GCG, Ser and Ala were removed and 8 μM Phe^I^-tRNA^AsnE2^ was added. For LC/MS analysis, 0.5 μM synthetic internal control peptide was added, whose sequence is ^Ac^PYYDYYDKKDYKDDDDK.

### LC/MS analysis of translated peptides

5 μl methanol was added to the 5-μl translation mix and centrifuged at 13 000 rpm, 25°C for 3 min. The supernatant was mixed with the same volume of 1% (v/v) TFA and centrifuged at 13 000 rpm, 25°C for 3 min. 7.5 μl of the supernatant was analyzed by LC/MS using Xevo G2-XS QTof system (Waters). A linear gradient from 1% B to 60% B was applied to a reverse-phase column (ACQUITY UPLC BEH C18, 1.7 μM, 2.1 × 150 mm, Waters), where Buffer A is water containing 0.1% (v/v) formic acid and buffer B is acetonitrile containing 0.1% (v/v) formic acid.

### MALDI-TOF MS analysis of translated peptides

Translated peptides were desalted with SPE C-tip (Nikkyo Technos) and co-crystalized with α-cyano-4-hydroxycinnamic acid on a sample plate. MALDI-TOF MS and MS/MS analyses were performed by UltrafleXtreme (Bruker Daltonics). A peptide calibration standard II (Bruker Daltonics) was used for external mass calibration.

## RESULTS

### Comprehensive analysis of the misdecoding pattern for 61 sense codons

**Figure 1.**
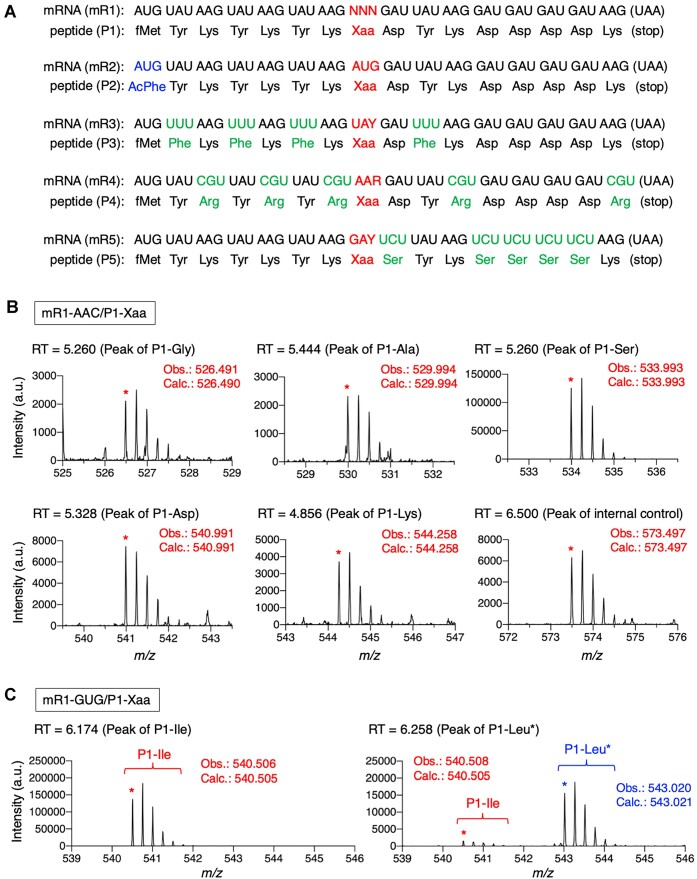
Evaluation of misdecoding susceptibility for 61 sense codons. (**A**) Sequences of mRNAs and corresponding peptides used for evaluation of misdecoding. Codons indicated by red were made vacant by removing corresponding amino acids from the translation system. AcPhe was introduced at AUG codon of mR2 by genetic code reprogramming. (**B**) Detection of peptides translated from mR1-AAC by LC/MS. Mass spectra of P1-Gly, P1-Ala, P1-Ser, P1-Asp and P1-Lys are shown. Mass spectrum of internal control peptide is also shown at right bottom. Obs. and Calc. indicate observed and calculated values of [M + 4H]^4+^. RT: retention time (min). (**C**) Detection of peptides translated from mR1-GUG by LC/MS. Mass spectra of P1-Ile and [D_10_]-labeled P1-Leu* are shown. Translation was carried out in the presence of [D_10_]-Leu in place of unlabeled Leu.

To evaluate the misdecoding pattern, we utilized a reconstituted *Escherichia coli in vitro* translation system, referred to as the FIT system (21), where an arbitrary amino acid, out of the 20 canonical ones, was removed to make a vacant codon. By using the FIT system including 19 amino acids (19-aa FIT system), we performed translation of an mRNA bearing a vacant NNN codon, which is cognate to the omitted amino acid. For instance, mR1-AAC has a vacant AAC codon at the 8th position (Figure 1A, mR1 bearing AAC at NNN indicated by red). Translation of mR1-AAC in the 19-aa FIT system lacking Asn induces misdecoding at the AAC codon. As shown in Figure 1B, misincorporation of Gly, Ala, Ser, Asp, and Lys into P1 peptide at the AAC codon was detected by LC/MS analysis (P1-Gly, P1-Ala, P1-Ser, P1-Asp and P1-Lys, respectively). Misincorporation of other amino acids into mR1-AAC was not detected. Identities of these products were confirmed by their *m/z* values and retention times. We confirmed that the difference in retention times between the product and the authentic sample was less than 0.02 min (Supplementary Table S2 and Supplementary Figure S2). Since mR1 has Met, Tyr, Lys and Asp, it is not possible to make the codons of these amino acids vacant. Therefore, for evaluation of Met AUG, Tyr UAY, Lys AAR and Asp GAY codons, mR2, mR3, mR4 and mR5 were used instead of mR1 (Figure 1A and Supplementary Table S3). In translation of mR2, *N*-acetyl-l-phenylalanine (AcPhe) was introduced at the N-terminus in place of fMet by using precharged AcPhe-tRNA^ini^ by means of flexizyme (Supplementary Figure S3A) (18). Since P1-Leu and P1-Ile have identical *m/z* values and close retention times, 6.190 min and 6.274 min, respectively, it is difficult to separate two peaks derived from P1-Leu and P1-Ile. Therefore, either Leu or Ile was replaced by isotope-labeled [D_10_]-Leu or [^13^C_6_]-Ile if either P1-Leu or P1-Ile was detected (Supplementary Table S3). For instance, translation of mR1-GUG was performed in the presence of [D_10_]-Leu (Leu*) instead of unlabeled Leu, where the peak of [D_10_]-labeled P1-Leu* could be clearly distinguished from that of P1-Ile owing to their different *m/z* values (Figure 1C).

**Figure 2.**
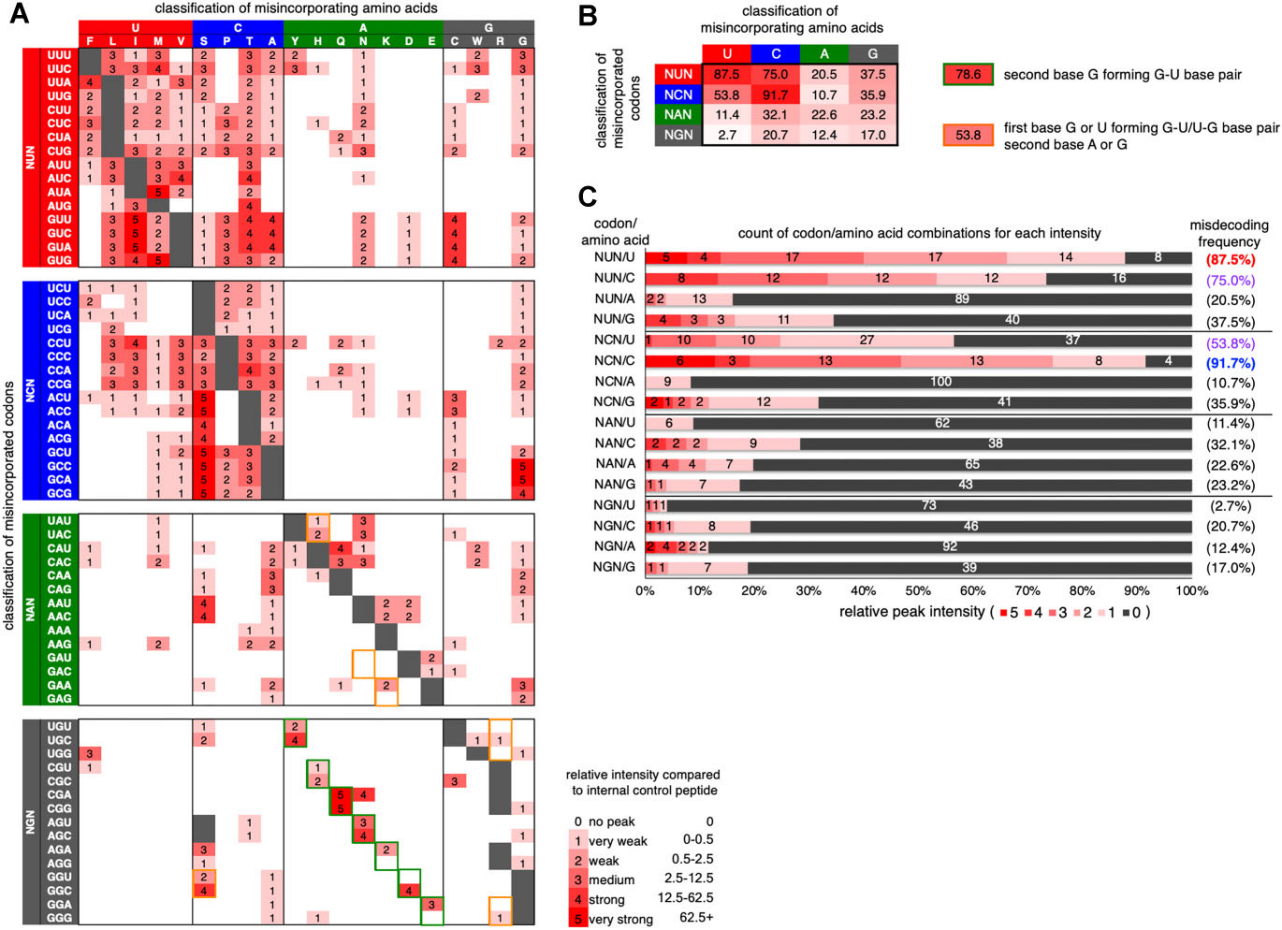
Misdecoding pattern for 61 sense codons against 19 noncognate amino acids. (**A**) Intensities of peptides bearing misincorporation of designated amino acids. Codons evaluated for misdecoding are shown at the left and misincorporating amino acids at the top. Cognate codon-amino acid combinations are indicated by grey, which were not tested for evaluation of intensity. Intensity is indicated by 1–5, which is estimated by relative peak intensity of each peptide compared to that of internal control peptide. Intensity 0, where no peak was detected, is indicated by white (number is not shown). Green boxes indicate the codon second base G forming a G-U base pair with the anticodon second base. Orange boxes indicate that the codon first base G or U forms a G-U base pair with the anticodon third base and that the codon second base is A or G. (**B**) Misdecoding frequencies for 16 combinations of codon/amino acid groups (left) and combinations that form G-U base pairs (right). Ratio of codon/amino acid combinations with intensities of 1–5 in each group was estimated as misdecoding frequency. For instance, in the case of NUN/U, 56 codon/amino acid combinations exhibited intensities of 1–5 out of 64 possible combinations, resulting in 87.5% misdecoding frequency (56/64). (**C**) Count of codon/amino acid combinations for each intensity for 16 combinations of codon/amino acid groups.

**Figure 3.**
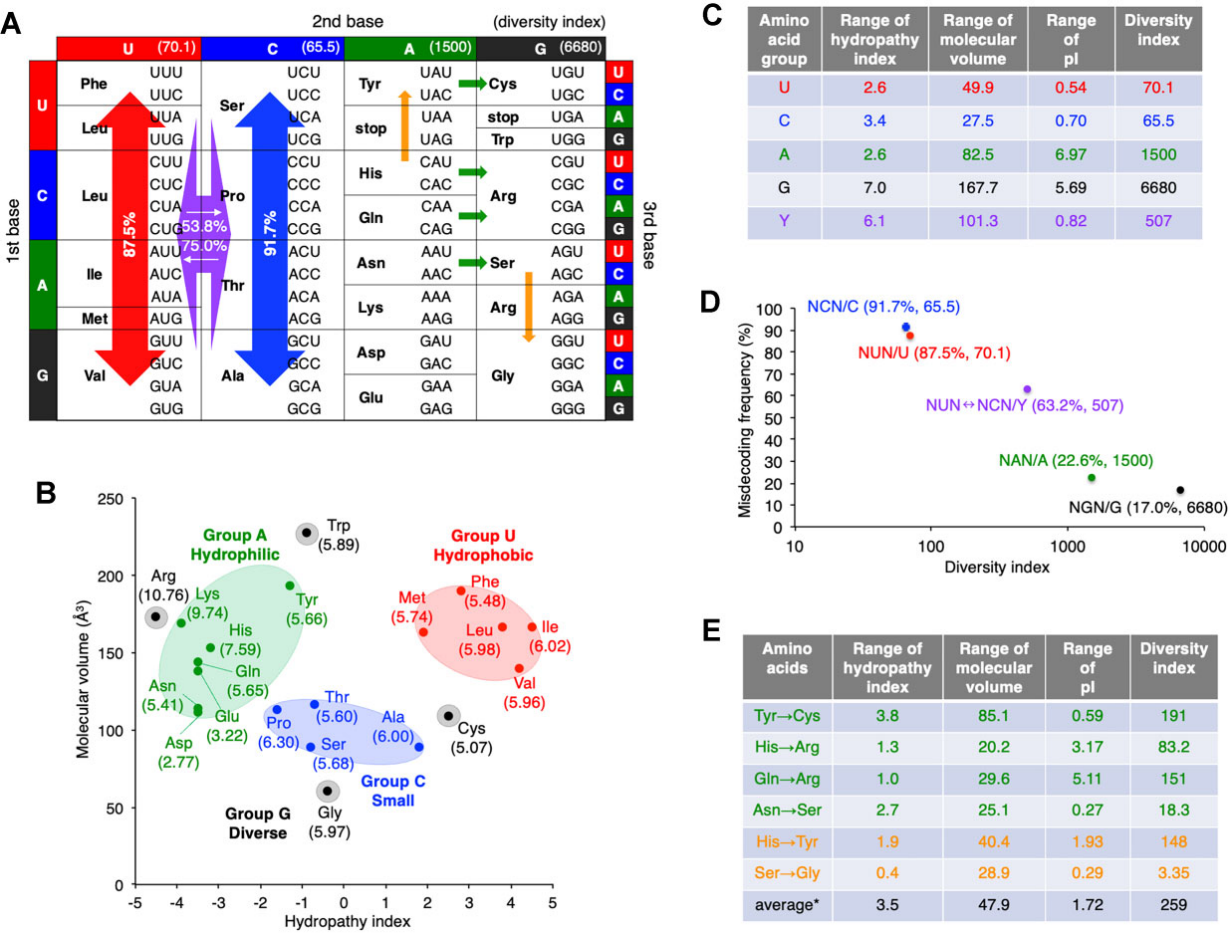
Negative correlation between misdecoding frequency and diversity of amino acids. (**A**) Direction of misdecoding in the codon table. Directions of misdecoding with high frequencies are indicated by arrows. Red and blue arrows indicate frequent misdecoding in a vertical direction at NUN and NCN codons. Purple arrow indicates misdecoding in a horizontal direction between NUN and NCN codons. Green and orange arrows indicate misdecoding caused by G-U base pair formation at either the first and second position. Misdecoding with > 50% frequencies are shown. (**B**) Evaluation of functional and structural features of 20 canonical amino acids. Hydropathy index and molecular volume are indicated at X- and Y-axes, respectively. Numbers in parentheses indicate pI values of amino acids. (**C**) Estimation of diversity index for each amino acid group. Group Y is a sum set of groups U and C. Numbers in parentheses indicate the numbers of stop codons included in the NAN and NGN codons. (**D**) Negative correlation between misdecoding frequency of codons and diversity index of amino acids. Values for combinations of NUN/U, NCN/C, NAN/A and NGN/G are indicated. (**E**) Estimation of diversity indexes for sets of two amino acids. Tyr→Cys, His→Arg, Gln→Arg, Asn→Ser, His→Tyr, and Ser→Gly. Arrows indicate the amino acids on the left are misincorporated in the codons on the right (For instance, Tyr→Cys shows misincorporation of Tyr into Cys UGY codons). * indicates the average value for all combinations of the 20 canonical amino acids.

Since a synthetic internal control peptide was added to the translation system, a peak derived from the internal control was also detected in the LC/MS analysis (Figure 1B, bottom right). Relative peak intensities of all misincorporation products observed for 61 sense codons were standardized by that of internal control and summarized in Figure 2A and Supplementary Figure S4. For instance, in the case of misincorporation at AAC codon of mR1-AAC, relative intensities of P1-Ser, P1-Ala, P1-Lys, P1-Asp and P1-Gly were 4, 1, 2, 2 and 1, respectively. Numbers of amino acids that were misincorporated into each codon were summarized in Supplementary Figure S5, where codons were classified into four groups, NUN, NCN, NAN and NGN, based on the type of nucleotide at the second position. The average numbers of misincorporating amino acids at NUN, NCN, NAN and NGN were 9.4, 7.6, 4.2 and 2.3, respectively, showing higher misdecoding frequencies at NUN and NCN codons than at NAN and NGN (Supplementary Figure S5, bottom). Misincorporating amino acids were also classified into four groups, U, C, A and G, based on the second nucleotide of the cognate codon (Group U: Phe, Leu, Ile, Met and Val, Group C: Ser, Pro, Thr and Ala, Group A: Tyr, His, Gln, Asn, Lys, Asp and Glu, and Group G: Cys, Trp, Arg and Gly). Notably, codon/amino acid combinations of NUN/U and NCN/C exhibited very high misdecoding frequencies (Figure 2, 87.5% and 91.7%, respectively), indicating that misincorporation of amino acids assigned at particular NUN (or NCN) codons frequently occurred at other NUN (or NCN) codons, e.g. misincorporation of Met (AUG) to GUU codon. This tendency is represented by red and blue vertical arrows in the codon table shown in Figure 3A (red arrow represents NUN/U; and blue arrow does NCN/C). Such vertical misdecoding is caused by nucleotide mismatches at the first and/or third position(s), where any mismatches are allowed, i.e. all of U, C, A and G can be paired with any nucleotide (U, C, A, G↔U, C, A, G). We also noticed that the combinations of NUN/C and NCN/U showed relatively high misdecoding frequencies (Figure 2, 75.0% and 53.8%, respectively). These misdecoding patterns include mismatches at the second position and are represented by a purple arrow in the codon table (Figure 3A). It should be noted that such frequent misdecoding in horizontal/diagonal directions is limited between NUN and NCN columns, where only U-G and C-A mismatched pairs are tolerated between codon second base and anticodon second base. NAN and NGN columns are not involved in this type of misdecoding, indicating limited misdecoding at codon second position compared to that at the first position. The above misdecoding pattern is summarized as ‘rule 1’ as follows: If the second codon base is U or C, misdecoding is frequently observed with a mismatch(es) at the first and/or third base where any types of mismatch are allowed, whereas frequent misdecoding with second-base mismatches is induced by only U-G and C-A pair formation.

If the second codon base is A or G, where rule 1 does not apply, G-U and U-G base pair formation between the first codon base and the third anticodon base induced relatively frequent misdecoding (Figure 2A, B, 53.8%, indicated by orange boxes). Misdecoding with a G-U base pair at the second codon base is also frequently observed when the second codon base is G (Figure 2A, B, 78.6%, indicated by green boxes). However, if both of the first and second positions have G-U or U-G base pairs, misdecoding was not observed (0%). This tendency is summarized as ‘rule 2’ as follows: If the second codon base is A or G, the frequency of misdecoding increases with a G-U or U-G pair at either the first or second position.

For the codon-amino acid combinations to which rule 1 is applied, 191 out of 256 combinations caused misdecoding (74.6%). For those to which rule 2 is applied, 18 out of 27 caused misdecoding (66.7%). Thus, most of the frequent misdecoding patterns are explained by rule 1, whereas rule 2 is minor. The other combinations had a misdecoding probablity of only 17.9% (157 out of 876 cases).

### Correlation between misdecoding frequency and diversity of amino acids

We next evaluated structural and functional diversities of amino acids of the four groups, U, C, A and G (Figure 3). As shown in Figure 3B, amino acids of groups U and C can be classified into hydrophobic and small amino acids, respectively, and fall within a narrow range of hydropathy index, molecular volume and isoelectronic point (pI) (22–24). Therefore, the functional and structural diversities of group U and C are small, which is indicated by the diversity indexes calculated by (hydropathy index range) × (molecular volume range) × (pI range) (Figure 3C, 70.1 and 65.5 for group U and C, respectively). In contrast, the diversity indexes for group A and G are two orders of magnitude larger (Figure 3C, 1500 and 6680 for group A and G, respectively). Regarding group A, the ranges of hydropathy index and molecular volume are relatively narrow, but the pI range is broad. As for group G, all of these ranges are broad. As shown in Figure 3D, we observed a significant negative correlation between misdecoding frequency of each column and diversity index of misincorporating amino acids (codon/frequency/diversity: NUN/87.5%/70.1, NCN/91.7%/65.5, NAN/22.6%/1500 and NGN/17.0%/6680). Codons that cause frequent misdecoding, i.e. NUN and NCN, are assigned to less diverse amino acid groups, i.e. groups U and C, whereas codons that cause less misdecoding, NAN and NGN, are assigned to more diverse amino acid groups, A and G. Since misdecoding occurs not only in the vertical but also in the horizontal/diagonal directions for NUN and NCN columns, the diversity index for group Y, a sum set of groups U and C, was also evaluated (Figure 3C, D, 507 for group Y). This value, 507, is still smaller than those of groups A and G, indicating that misdecoding between NUN and NCN columns should be relatively tolerated. These results show robustness of the modern genetic code against the rule-1-type misdecoding.

To evaluate the deleterious effect caused by the rule-2-type misdecoding, functional and structural similarities of the following sets of amino acids were also estimated based on the diversity index: Tyr/Cys, His/Arg, Gln/Arg, Asn/Ser, His/Tyr and Ser/Gly (Figure 3E, 191, 83.2, 151, 18.3, 148 and 3.35, respectively). Based on rule 2, significant misincorporation of Tyr, His, Gln and Asn into Cys (UGY), Arg (CGY), Arg (CGR) and Ser (AGY) codons, respectively, were induced by G-U or U-G pairs at codon second position (Figure 3A, indicated by green arrows), whereas misincorporation of His and Ser into Tyr (UAY) and Gly (GGY) codons induced by G-U or U-G pair formation at codon first position (Figure 3A, indicated by orange arrows). Since all of these diversity index values were smaller than the average of all amino acid combinations (Figure 3E, 259), deleterious effects caused by these misdecoding patterns would be restrained. These results indicate that functionally and structurally similar amino acids are assigned at adjacent codon boxes in NAN and NGN columns so that deleterious effects of misdecoding caused by G-U/U-G base pair formation can be minimized.

### Correlation between misdecoding frequency and amino acid replacement frequency in proteins during evolution

During evolution, proteins are subjected to amino acid replacements in their primary sequences, which are acquired through the processes of natural selection. Here, we analyzed the correlation between misdecoding frequency in translation and amino acid replacement frequency in proteins caused by natural selection. The amino acid replacement frequency can be evaluated by using PAM (point accepted mutation) matrix, which was established by Dayhoff et al (25). Supplementary Figure S6A shows a rearranged PAM30 matrix, in which the 20 canonical amino acids are classified into four groups, U, C, A and G, following the same classification rule as Figure 2A based on the second nucleotide of the corresponding codon (Group U: Phe, Leu, Ile, Met and Val, Group C: Ser, Pro, Thr and Ala, Group A: Tyr, His, Gln, Asn, Lys, Asp and Glu and Group G: Cys, Trp, Arg and Gly). The scores in this matrix represent the mutation probability during evolution for designated amino acid pairs, where higher scores indicate higher probability. Supplementary Figure S6B summarizes the average PAM scores for the four groups: U, C, A and G. The average of all scores is −6.5. As for the mutations within the same groups, group C has the highest average score (−1.5), followed by group U (−1.9), group A (−3.6) and group G (−9.7). This order is identical to that of misdecoding frequency (Figure 2B, group/frequency: C/91.7%, U/87.5%, A/22.6% and G/17.0%), showing the correlation between misdecoding frequency in translation and mutation frequency during evolution. As the PAM scores also indicate how tolerable a replacement is with respect to retention of protein function, we concluded that misdecoding more frequently occurs at tolerable amino acid pairs. As for the mutations between different groups, U↔C mutations exhibited a slightly higher score (−6.1) than the average (−6.5); however, this difference is too small to conclude that U↔C mutations are significantly preferred to the other combinations. This is likely because such mutations between different groups require more nucleotide substitutions in codon (1, 2 or 3 substitutions) than the mutations within the same groups (limited to 1 or 2), and thus they should be less pronounced in the PAM matrix.

### Correlation between the number of mismatches and misdecoding frequency

**Figure 4.**
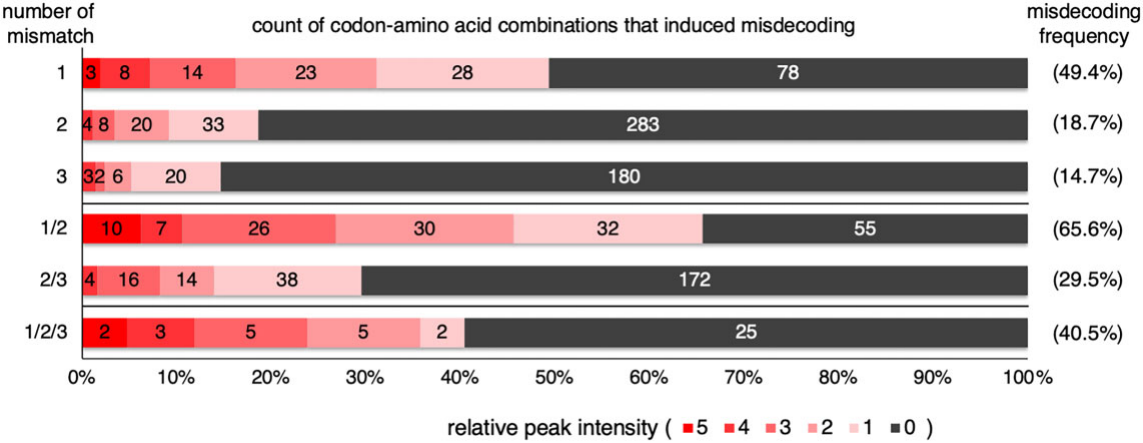
Correlation between misdecoding frequency and number of mismatches. Numbers of mismatches between codon and anticodon are classified into six patterns, 1, 2, 3, 1/2, 2/3 and 1/2/3. Counts of misdecoding for the 6 mismatch patterns are summarized.

We next evaluated the number of mismatches between codon of mRNA and anticodon of tRNA, and its effect on the misdecoding frequency. Since non-Watson-Crick base pair formation is generally allowed between the third codon base and the first anticodon base, such pairs are considered as matched pairs in this analysis. Supplementary Figure S7 summarizes the previously reported codon-anticodon interactions and nucleotide modifications at anticodon first base in *E. coli* (26–34). The codon-anticodon combinations that induced misdecoding were shown in Supplementary Table S4. For instance, cmo^5^UAC anticodon of tRNA^Val1^ is able to decode GUU, GUC, GUA and GUG codons. Therefore, base pair formations between cmo^5^U at the first anticodon base and any nucleotide at the third codon base are considered as matched pairs. In the case of Val misincorporation at UUA codon, two anticodon sequences of tRNA^Val^ isoacceptors, GAC and cmo^5^UAC, are available. The first codon base, U, is mismatched with the third anticodon base, C (Supplementary Figure S8, indicated by +). The second codon base, U, is matched with the second anticodon base, A (Supplementary Figure S8, indicated by −). The third codon base, A, is matched with cmo^5^U but mismatched with G at the first anticodon base (Supplementary Figure S8, indicated by ±). In total, the number of mismatches is 1 or 2 in this case, which is not uniquely determined (Supplementary Figure S8, indicated by 1/2). In the same way, the numbers of mismatches between codon and anticodon were counted for all misdecoding patterns (Supplementary Figure S8, 61 codons × 19 amino acids). Then, the numbers of mismatches were classified into six types, 1, 2, 3, 1/2, 2/3 and 1/2/3. Consequently, we discovered a negative correlation between the misdecoding frequency and the number of mismatches for classifications 1, 2 and 3 (Figure 4, 49.4%, 18.7% and 14.7% for 1, 2 and 3 mismatches), indicating that codon-anticodon combinations with fewer mismatches induced more frequent misdecoding. This is likely because combinations of mRNA and tRNA with fewer mismatches are generally more stable at the decoding center of the ribosome and therefore have higher efficiency for decoding than combinations with more mismatches. Similarly, classification 1/2 showed more frequent misdecoding than 2/3 (Figure 4, 65.6% and 29.5%, respectively). The reason why 1/2 and 2/3 showed higher misdecoding frequencies than 2 and 3, respectively, is attributed to the involvement of multiple isoacceptor tRNAs in inducing misdecoding in the case of 1/2 and 2/3. The same explanation could be applied to the high misdecoding frequency in 1/2/3 (40.5%).

In the case of 1 mismatch, only vertical or horizontal direction misdecoding occurs, whereas 2 and 3 mismatches possibly cause misdecoding in a diagonal direction in the codon table. It is remarkable that misdecoding occurs even if there are 3 mismatches between codon and anticodon, though the frequency is low (14.9%). 31 codon-anticodon combinations that have 3 mismatches induced misdecoding (Supplementary Table S5). Among them, 21 codons were classified into NUN and NCN columns, whereas only 10 into NAN and NGN columns, showing higher susceptibility of NUN and NCN to 3-mismatch misdecoding.

**Figure 5.**
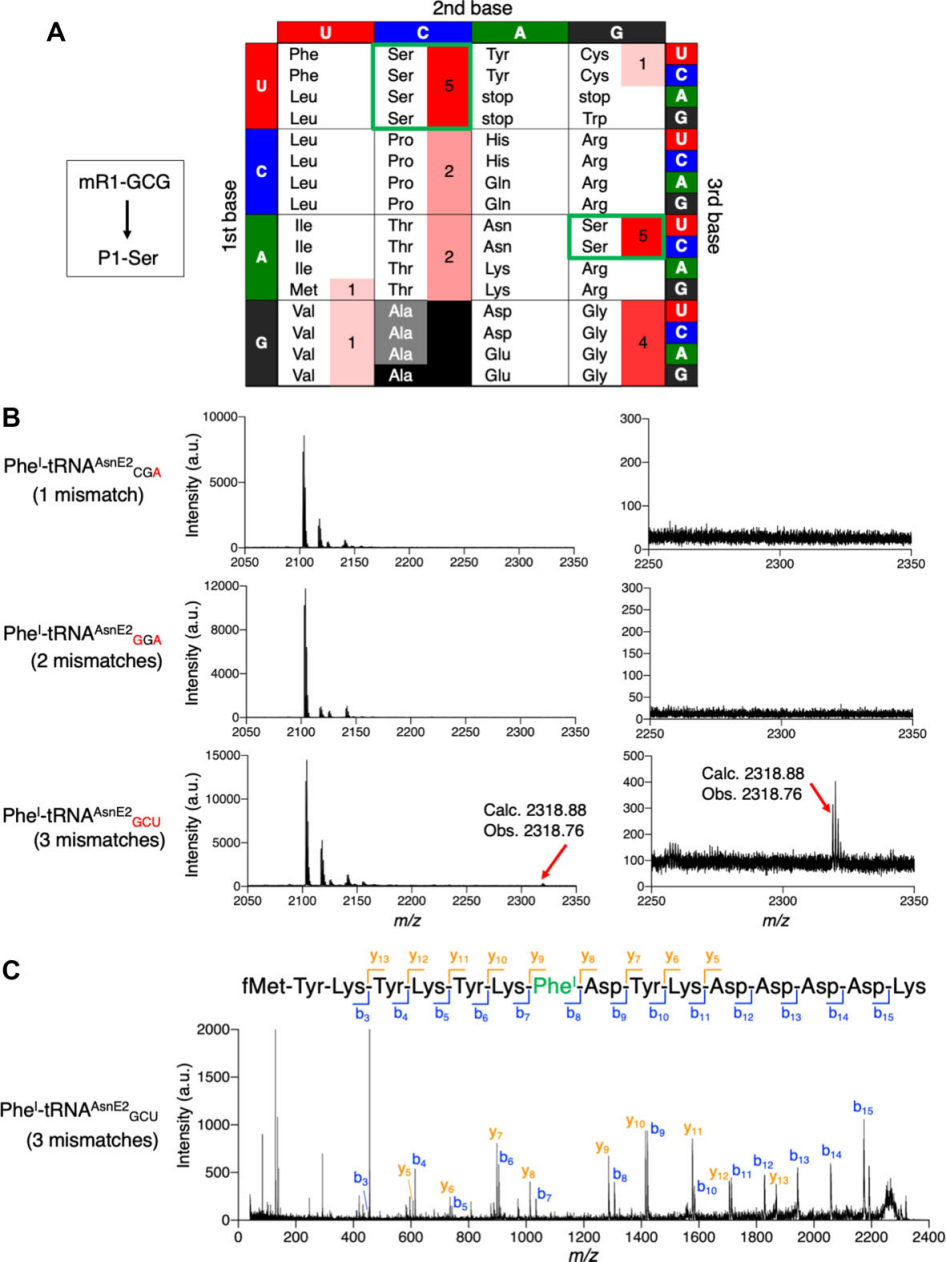
Misincorporation of Phe^I^ at GCG codon induced by anticodons of tRNA^Ser^ isoacceptors. (**A**) Misincorporation of noncognate amino acids at GCG codon of m1-GCG. Strong misincorporation of Ser at GCG codon was observed, which was possibly induced by any of the four tRNA^Ser^ isoacceptors bearing GGA, mcmo^5^UGA, CGA and GCU anticodons. (**B**) Induction of Phe^I^ misincorporation at GCG codon by using precharged Phe^I^-tRNA^AsnE2^ bearing CGA, GGA and GCU anticodons. Mismatched bases in anticodon are indicated by red. Translated peptides were analyzed by MALDI-TOF MS. Obs. and Calc. indicate observed and calculated values of [M + H]^+^. (**C**) MALDI-TOF MS/MS analysis of P1-Phe^I^. Phe^I^ was introduced at GCG codon of m1-GCG using Phe^I^-tRNA^AsnE2^_GCU_.

If there are multiple isoacceptor tRNAs that are able to decode a particular amino acid, it is not possible to determine which isoacceptor causes misdecoding. For instance, in the case of misincorporation of Ser to GCG codon, four tRNA^Ser^ isoacceptors that have GGA, mcmo^5^UGA, CGA and GCU anticodons are possibly involved, where all of 1, 2 and 3 mismatches are possible (Figure 5A). In order to examine which anticodon induces misdecoding, we performed translation of mR1-GCG in the absence of Ala and Ser, where precharged 4-iodophenylalanyl-tRNA^AsnE2^ (Phe^I^-tRNA^AsnE2^) bearing CGA, GGA or GCU anticodon was supplemented. Note that tRNA^AsnE2^ is an engineered tRNA generally used for genetic code reprogramming and that mcmo^5^UGA anticodon was not tested due to *in vitro* transcription of tRNA^AsnE2^ (Supplementary Figure S3B). Consequently, Phe^I^-tRNA^AsnE2^ bearing CGA and GGA anticodons did not induce misincorporation of Phe^I^ (Figure 5B, 1 and 2 mismatches, respectively), whereas Phe^I^-tRNA^AsnE2^ bearing GCU anticodon induced Phe^I^ misincorporation (Figure 5B, 3 mismatches). We also confirmed that Phe^I^ was introduced at the GCG codon using Phe^I^-tRNA^AsnE2^_GCU_ by MALDI-TOF MS/MS (Figure 5C). Since this system employed orthogonal tRNA^AsnE2^ for incorporation of nonproteinogenic Phe^I^, we can exclude a possibility that the misincorporation is caused by misacylation. Therefore, these results validated that misdecoding occurs even if there are 3 mismatches between codon and anticodon and even competes with 1- or 2-mismatch misdecoding when multiple isoacceptors are available.

## DISCUSSION AND CONCLUSION

In this study, we proposed two rules that describe the misdecoding pattern. Based on rule 1, misdecoding frequently occurs in a vertical direction induced by a mismatch(es) at the first and/or third base of codon. In contrast, misdecoding in a horizontal direction, induced by a second-base mismatch, is relatively limited. Therefore, it is reasonable that the second base, rather than the other bases, was first used for discrimination of amino acids in the primordial 4-column genetic code (Supplementary Figure S1, top). The third codon base is less robust than the first base against misdecoding because non-Watson-Crick base pairs are widely tolerated (Supplementary Figure S7). Thus, the first base, rather than the third base, was utilized for expansion of the 4-columun code into the 16-supercodon code (Supplementary Figure S1, middle). These results support the relevance of the 2–1–3 model in genetic code evolution.

Rule 1 also shows that NUN and NCN columns suffer from more severe vertical misdecoding than NAN and NGN. We evaluated the diversity index of amino acids assigned to each column and showed that functionally and structurally less diverse amino acids are assigned to NUN and NCN, whereas more diverse ones are assigned to NAN and NGN (Figure 3C). Since misdecoding in horizontal/diagonal directions also occurs between NUN and NCN codons to a certain degree, we also evaluated the diversity index for amino acids assigned to NYN column, which was still smaller than those of amino acids assigned to NAN and NGN. We also estimated diversity indexes for pairs of amino acids (cognate amino acid and misincorporating amino acid) involved in misincorporations caused by G-U or U-G base pair formation, on the basis of rule 2. All of the diversity indexes of these sets are smaller than the average value for all amino acid combinations (Figure 3E). This observation supports the experimental results in several preceding reports that showed frequent misincorporation caused by G-U and U-G mismatches (16,35,36). Rozov and coworkers analyzed crystal structures of 70S ribosome complexes with noncognate mRNA-tRNA pairs, where G-U and U-G pairs at the first or second position of codon mimic the Watson-Crick geometry (37,38). Compared to other types of mismatches, G-U and U-G pairs form more stable geometry. C-A pair is also able to adopt Watson-Crick geometry, although its stability is much lower than those of G-U and U-G. In our result, C-A mismatch at the codon second position induced misincorporation of group U amino acids into NCN codons with relatively high frequency (Figure 2B, 53.8%).

In an empirical hypothesis proposed in 1960s, transversion misdecoding is less frequent than transition misdecoding and purine misdecoding is less frequent than that of pyrimidine (7,39,40). However, conclusive experimental data to validate this idea have not been provided for a long time. This hypothesis is not fully consistent with our result, where all mismatch patterns are tolerated at the first codon base if the second base is C or U. Even if the second codon base is A or G, G at the first or second position causes frequent misdecoding by a G-U base pair formation. The preceding studies analyzed only limited numbers of selected codons for misdecoding and therefore were not sufficient to reveal this tendency. In this study, a comprehensive, quantitative, and statistical analysis of misdecoding patterns for 61 sense codons was performed to provide more conclusive and reliable rules for misdecoding pattern, which enables precise evaluation of selective pressure applied to genetic code evolution.

Although not only misdecoding but also aminoacylation error, transcription error and replication error can cause amino acid misincorporation, preceding studies suggest that most misincorporation is caused by misdecoding (15,16). Thus, misdecoding is a possible selective pressure for genetic code evolution. The frequency of misdecoding differs depending on the type of codon-anticodon combination and the range has been estimated to be 10^−3^ to 10^−6^ per codon (35). Some G-U pairs exhibit high frequency of misincorporation above 10^−3^ per codon. On the other hand, those of aminoacylation error, transcription error and replication error are approximately 10^−4^–10^−5^, 10^−6^ and 10^−8^ per codon, respectively (41–43). As misacylation frequency is relatively close to misdecoding frequency, we cannot exclude the possibility that our result includes misincorporation caused by misacylation. However, Kramer *et al.* showed that translational error is mainly caused by misdecoding rather than misacylation (15). In their study, the level of Lys misincorporation at Arg AGR codons significantly decreased when the cognate tRNA^Arg^ was overexpressed, indicating that Lys misincorporation is caused by competition between Lys-tRNA^Lys^ and Arg-tRNA^Arg^ rather than misacylation of Lys on tRNA^Arg^. Later on, Zhang *et al.* also supported this conclusion in their report (16). Particularly in our study, the effects of transcription and replication errors may be disregarded because misincorporation was enforced by the removal of particular amino acids, i.e. in the complete absence of the aminoacyl-tRNAs by the use of a reconstituted translation system, referred to as FIT system. This system enables us to simulate primitive translation conditions where all components in the modern cellular translation system have not yet been evolved, e.g. those responsible for degrading cellular tRNAs to prevent mistranslation upon amino acid starvation in cells (44) and tmRNA-mediated degradation of peptide fragments (45). Thus, we could test a specific constraint of misdecoding event and ask how the genetic code could have evolved in a primitive translation system. Our result reported here should be of importance for discussion of a possible path(s) of the evolution of the genetic code because only a smaller set of amino acids could have been available in such a primitive translation system.

The data set obtained in this study will be beneficial to research in a broad range of fields, such as genetics, proteomics and chemical biology. For instance, genetic code reprogramming enables introduction of nonproteinogenic amino acids (nPAAs) in the codon table. However, introduction of nPAAs with low incorporation efficiencies, such as *N*-methyl amino acids, α-hydrazino acids, and α,α-disubstituted amino acids, causes severe misincorporation due to competition of noncognate aminoacyl-tRNAs, making it difficult to express full-length peptides/proteins bearing desired nPAAs (46–48). This issue could be solved by optimizing codon assignment based on the rules proposed in this study. Undesired misincorporation could be circumvented by using more robust codons that cause less frequent misdecoding, such as GAU, for incorporation of inefficient nPAAs, whereas efficient substrates could be assigned to less robust codons. Such an effort would lead to creation of novel artificial genetic code with extensive reprogramming introducing a number of nPAAs in the codon table.

## DATA AVAILABILITY

The data underlying this research are available in the article and in its online supplementary material.

## SUPPLEMENTARY DATA

Supplementary Data are available at NAR Online.

## Supplementary Material

gkad707_Supplemental_Files
