# Supplementary material for: A comprehensive analysis of translational misdecoding pattern and its implication on genetic code evolution
Source: Nucleic Acids Res. 2023 Aug 28;51(19):10642–52. doi: 10.1093/nar/gkad707 (PMC10602915; doi:10.1093/nar/gkad707)
Supplement: gkad707_Supplemental_Files [file gkad707_supplemental_files.zip › Supplementary Figures.pdf]

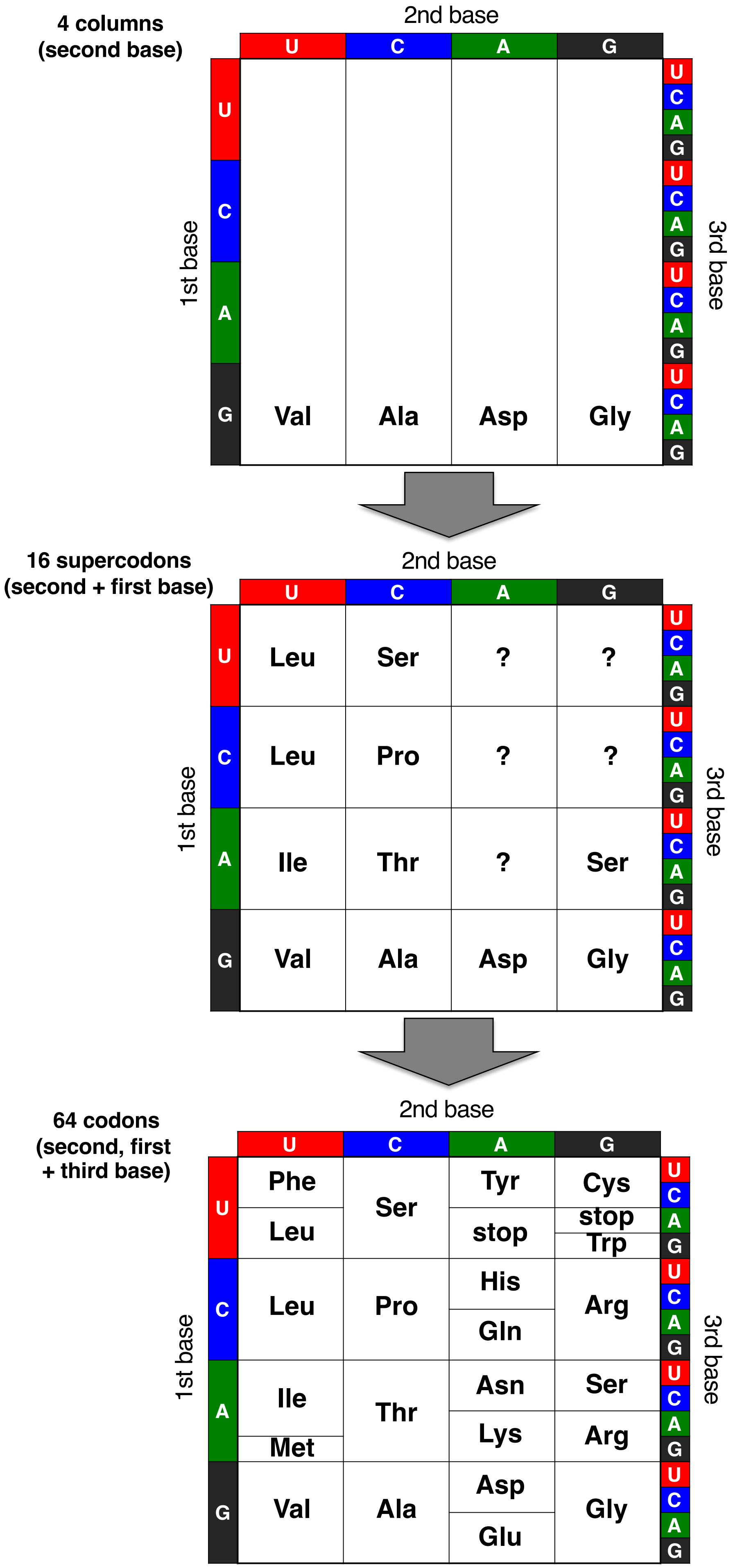

Supplementary Figure 1. Evolution of genetic code in 2-1-3 model. The four simplest amino acids, Val, Ala, Asp and Gly, were first assigned at NUN, NCN, NAN and NGN columns, respectively, using the second base of codon for discrimination. Then, the code was expanded into 16 supercodons using the first base to assign Leu, Ile, Ser, Pro and Thr. Finally, the third base was utilized to divide the code into the current 64 codons, where canonical 20 amino acids were assigned.

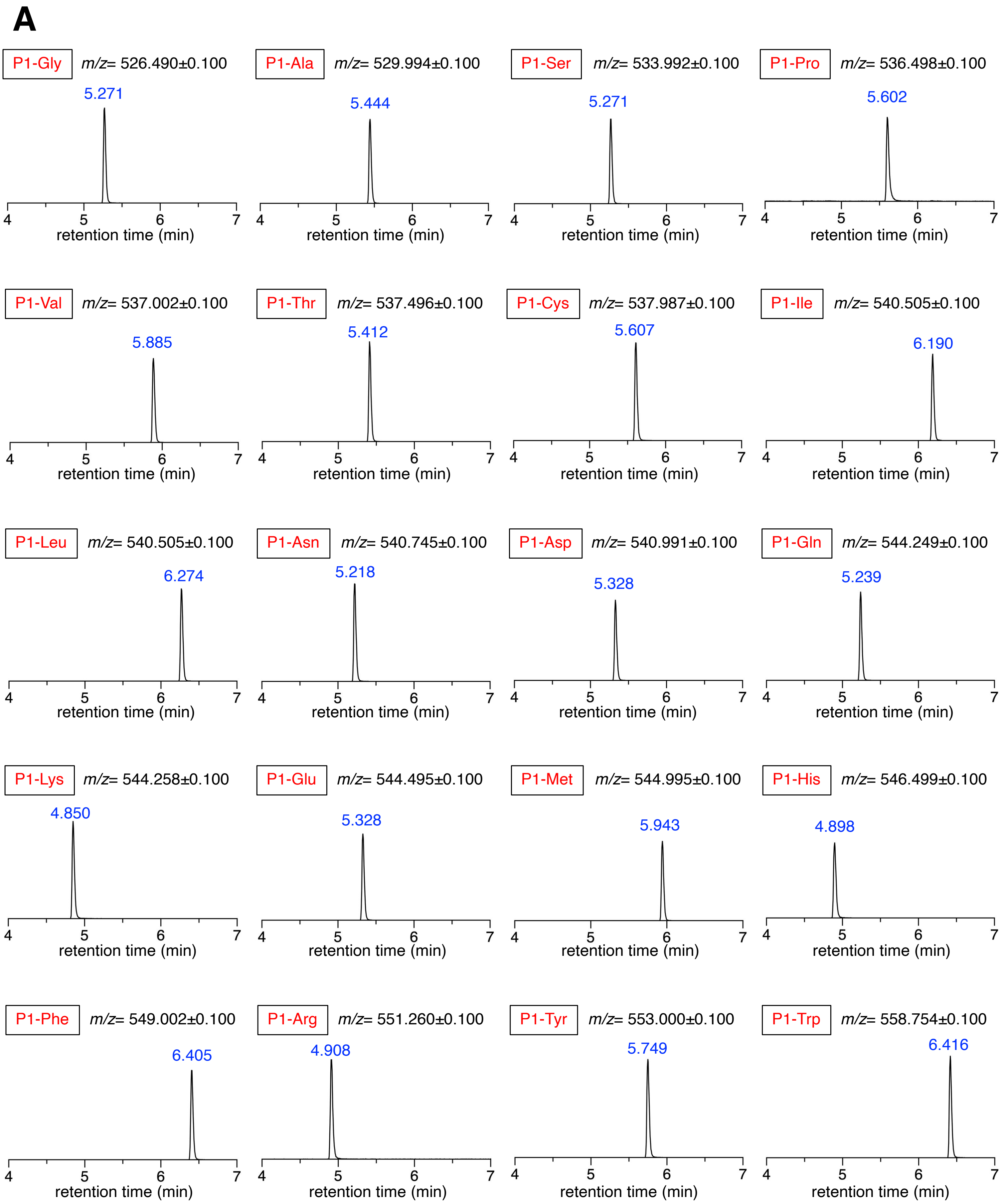

Supplementary Figure 2. LC/MS analysis of authentic peptides. Authentic peptides derived from P1 (A), P2 (B), P3 (C), P4 (D) and P5 (E). Range of *m/z* values of $[M+4H]^{4+}$ is indicated at the top. Retention time of each peptide is shown by blue.

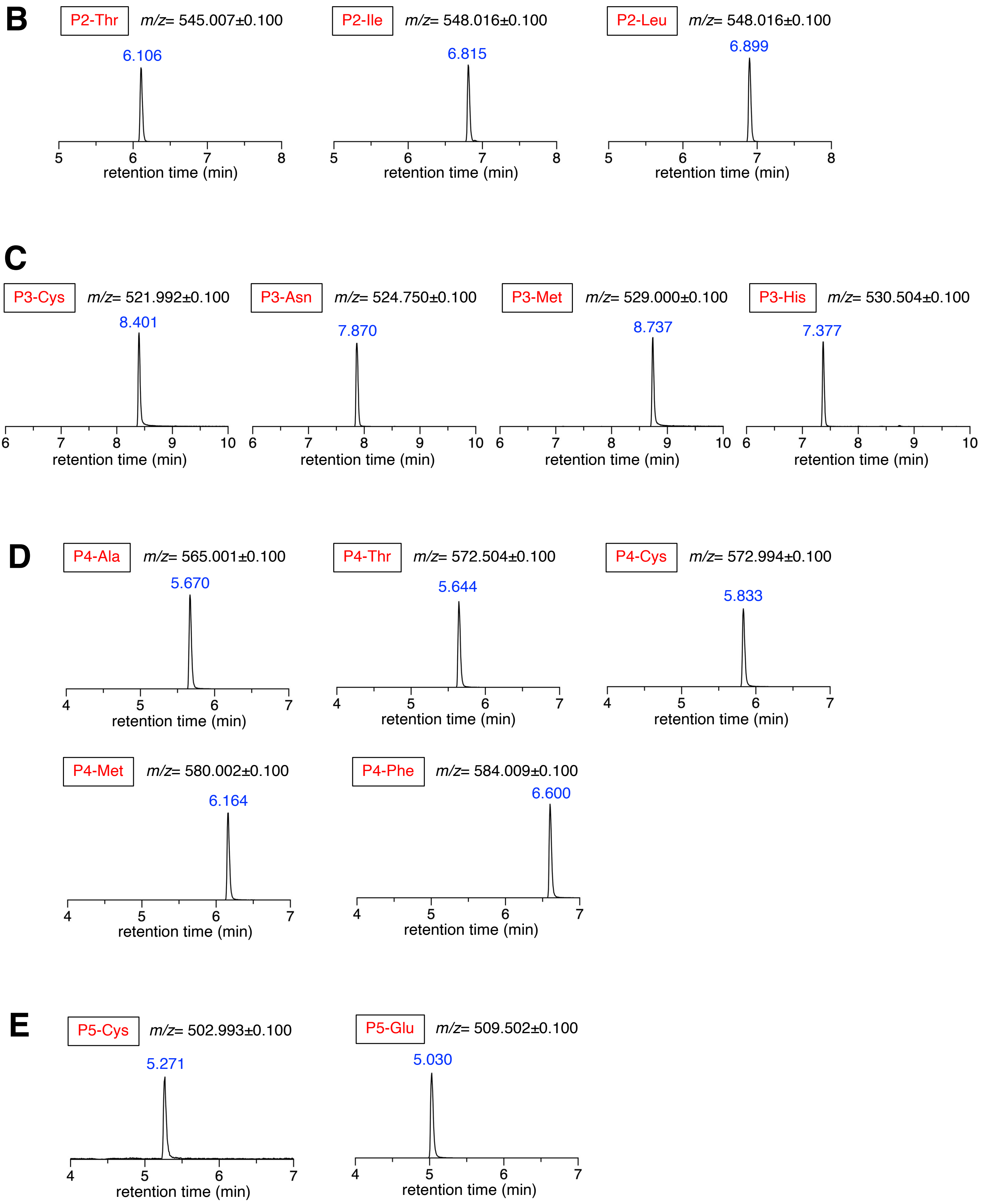

Supplementary Figure 2 continued.

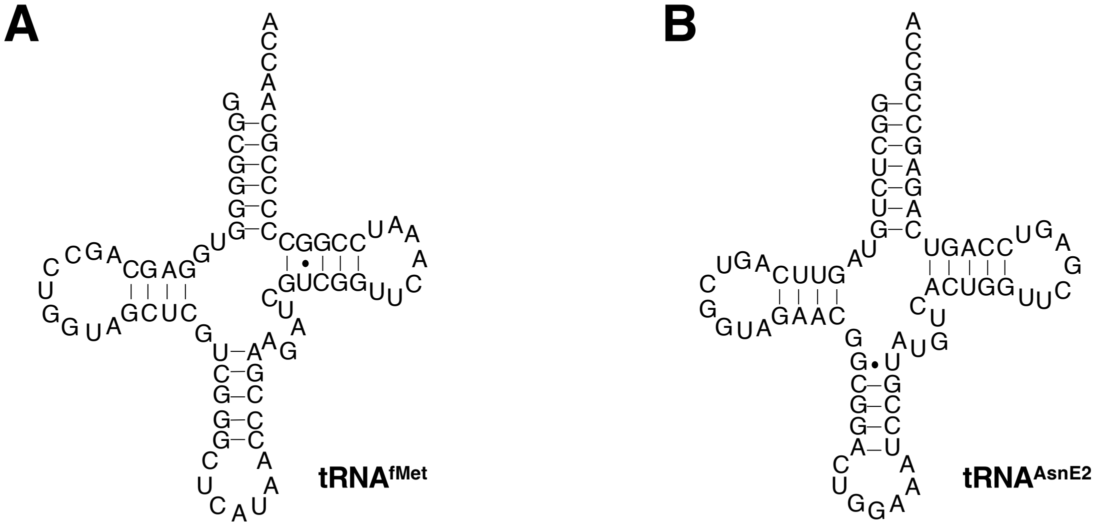

Supplementary Figure 3. Secondary structures of tRNA^{fMet} and tRNA^{AsnE2} used for genetic code reprogramming. (A) tRNA^{fMet} used for incorporation of AcPhe at the N-terminus of mR2. The 5'-terminal C of wild-type *E. coli* tRNA^{fMet} was replaced with G to improve transcription efficiency. **(B)** tRNA^{AsnE2} used for incorporation of Phe^{I} in elongation. The anticodon loop sequence was changed accordingly to read cognate codons. See also Supplementary Table 1 for the sequence.

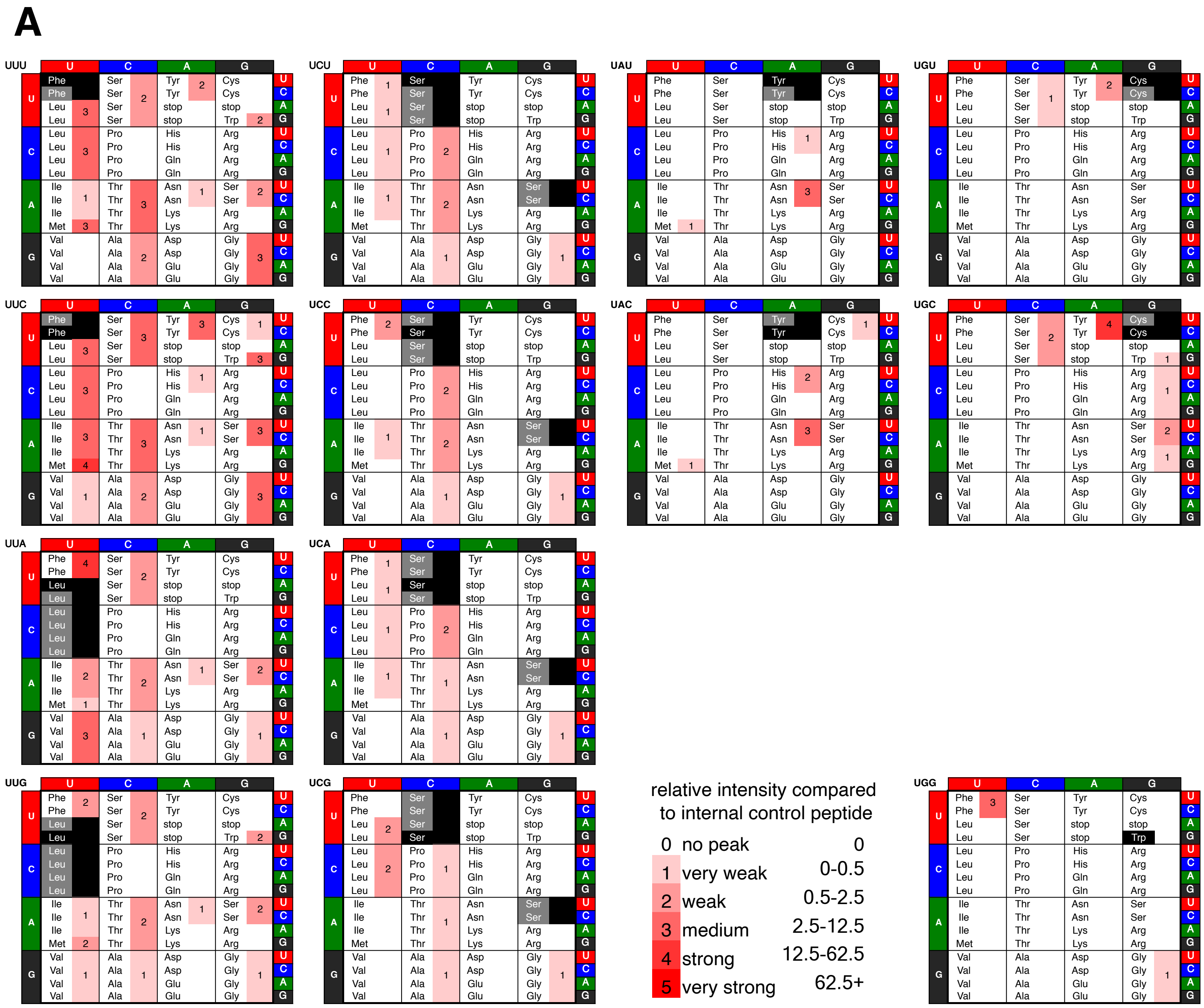

Supplementary Figure 4. Misdecoding patterns for 61 sense codons. Intensities of peptides bearing misincorporation of designated amino acids. Codons evaluated for misdecoding are indicated at the top left of the table. Intensity is indicated by 0–5, which is estimated by relative peak intensity of each peptide compared to that of internal control peptide. (A) UNN codons, (B) CNN codons, (C) ANN codons and (D) GNN codons.

B

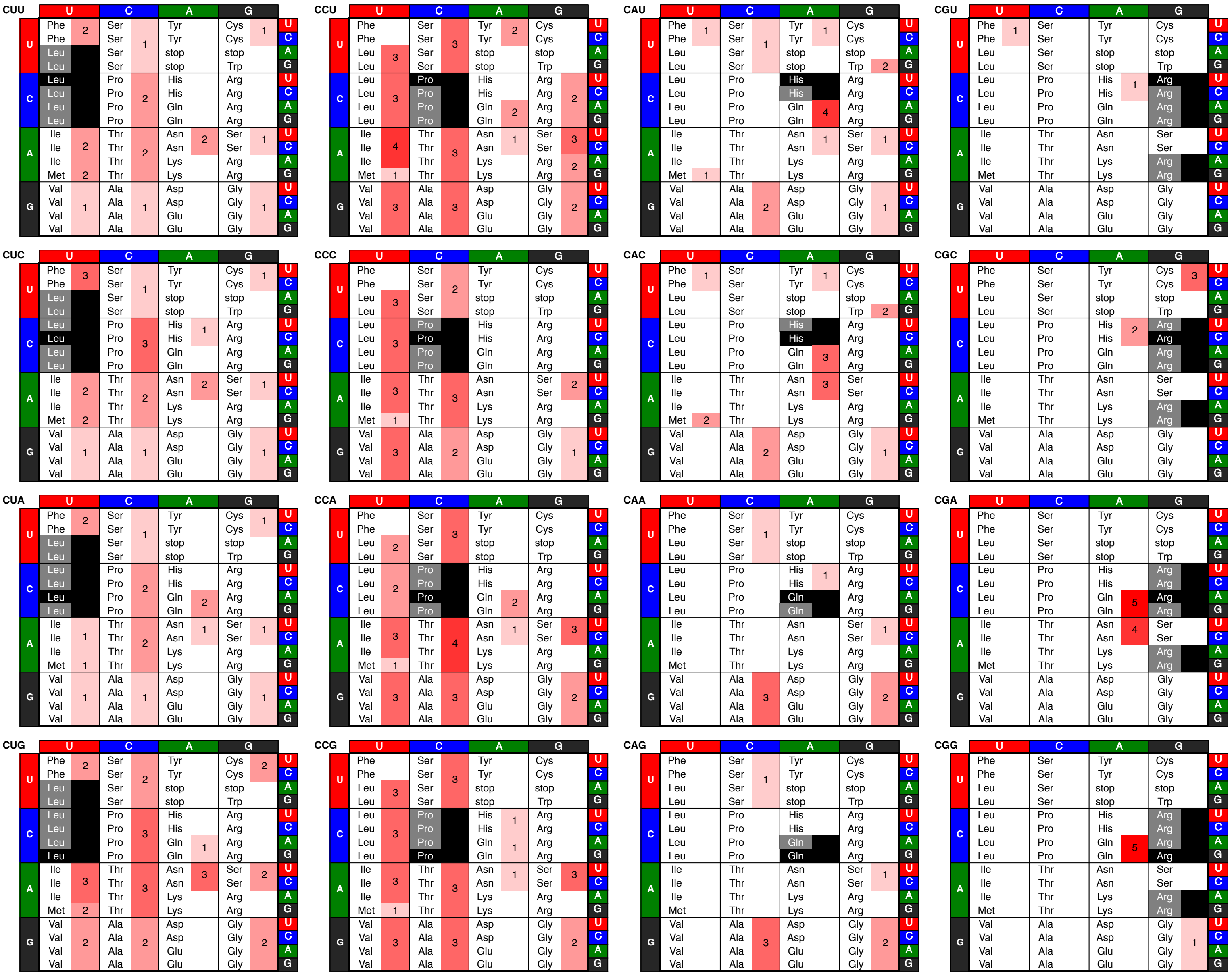

Supplementary Figure 4 continued.

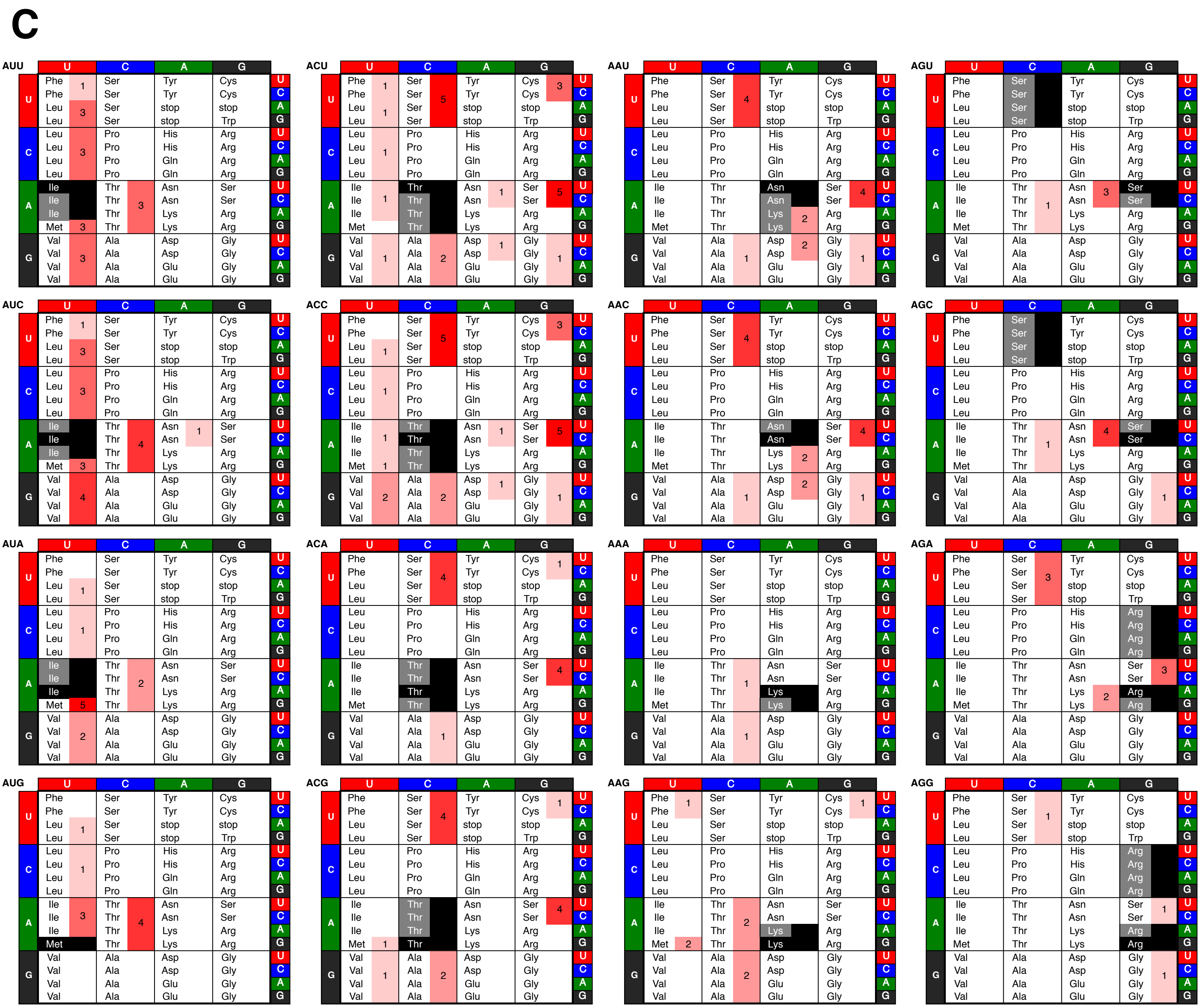

Supplementary Figure 4 continued.

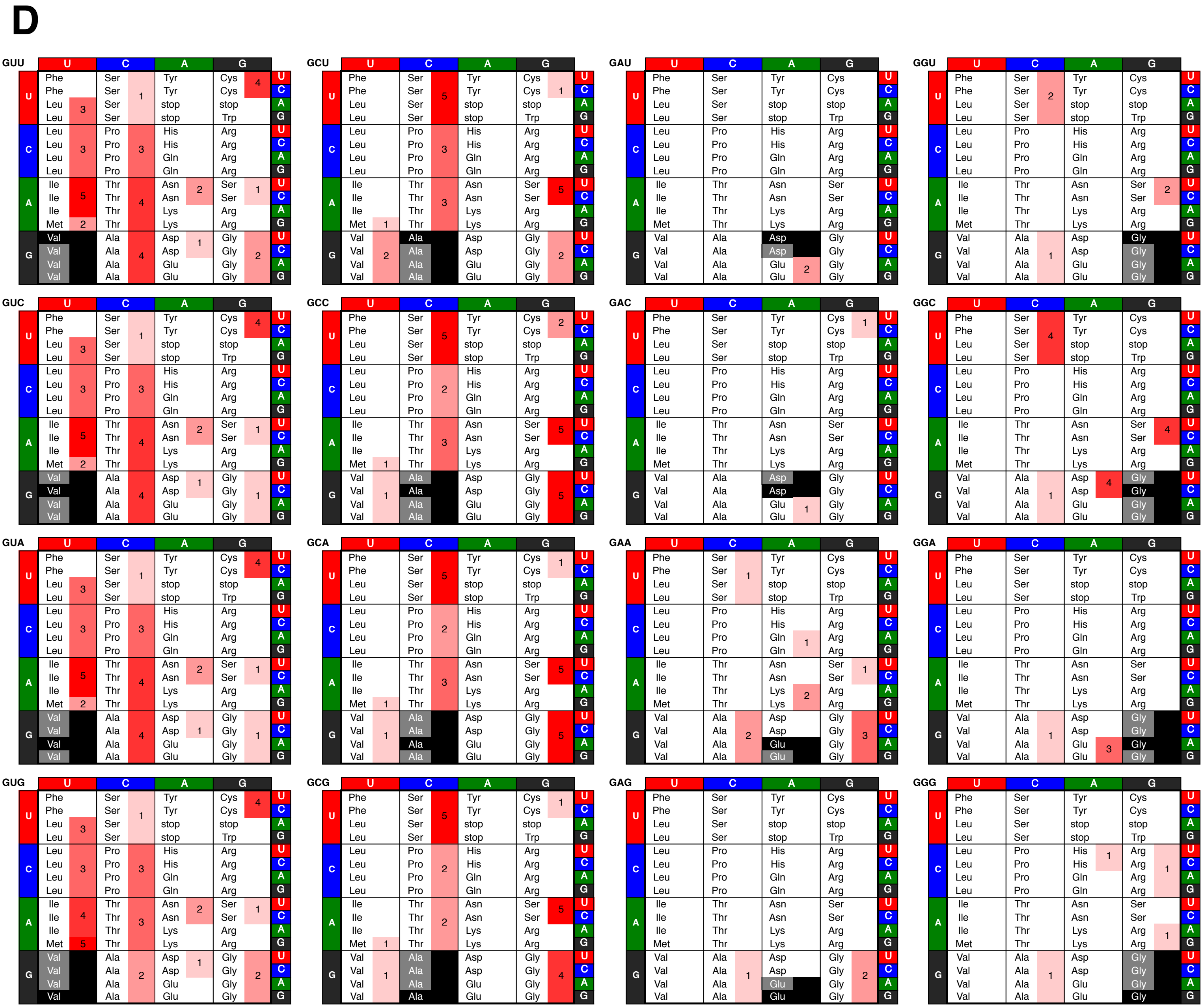

Supplementary Figure 4 continued.

1st base	2nd base: U		C		A		G		3rd base
U	Phe	10	Ser	7	Tyr	3	Cys	2	U
U	Phe	13	Ser	6	Tyr	4	Cys	4	C
U	Leu	9	Ser	7	stop		stop		A
U	Leu	10	Ser	5	stop		Trp	2	G
C	Leu	11	Pro	12	His	9	Arg	2	U
C	Leu	12	Pro	8	His	8	Arg	2	C
C	Leu	12	Pro	10	Gln	4	Arg	2	A
C	Leu	12	Pro	11	Gln	3	Arg	2	G
A	Ile	5	Thr	10	Asn	5	Ser	2	U
A	Ile	6	Thr	10	Asn	5	Ser	3	C
A	Ile	4	Thr	3	Lys	2	Arg	2	A
A	Met	3	Thr	5	Lys	5	Arg	2	G
G	Val	11	Ala	7	Asp	1	Gly	2	U
G	Val	11	Ala	7	Asp	2	Gly	3	C
G	Val	11	Ala	7	Glu	5	Gly	2	A
G	Val	11	Ala	7	Glu	2	Gly	3	G
average		9.4		7.6		4.1		2.3	

Supplementary Figure 5. Numbers of amino acids that are misincorporated into the designated codons. Average values for NUN, NCN, NAN and NGN codons are shown in the bottom.

A

		U					C				A							G			
		F	L	I	M	V	S	P	T	A	Y	H	Q	N	K	D	E	C	W	R	G
U	F		-3	-2	-4	-8	-6	-10	-9	-8	2	-6	-13	-9	-14	-15	-14	-13	-4	-9	-9
	L	-3		-1	1	-2	-8	-7	-7	-6	-7	-6	-5	-7	-8	-12	-9	-15	-6	-8	-10
	I	-2	-1		-1	2	-7	-8	-2	-5	-6	-9	-8	-5	-6	-7	-5	-6	-14	-5	-11
	M	-4	1	-1		-1	-5	-8	-4	-5	-11	-10	-4	-9	-2	-11	-7	-13	-13	-4	-8
	V	-8	-2	2	-1		-6	-6	-3	-2	-7	-6	-7	-8	-9	-8	-6	-6	-15	-8	-5
C	S	-6	-8	-7	-5	-6		-2	0	0	-7	-6	-5	0	-4	-4	-4	-3	-5	-3	-2
	P	-10	-7	-8	-8	-6	-2		-4	-2	-13	-4	-3	-6	-6	-8	-5	-8	-14	-4	-6
	T	-9	-7	-2	-4	-3	0	-4		-1	-6	-7	-5	-2	-3	-5	-6	-8	-13	-6	-6
	A	-8	-6	-5	-5	-2	0	-2	-1		-8	-7	-4	-4	-7	-3	-2	-6	-13	-7	-2
A	Y	2	-7	-6	-11	-7	-7	-13	-6	-8		-3	-12	-4	-9	-11	-8	-4	-5	-10	-14
	H	-6	-6	-9	-10	-6	-6	-4	-7	-7	-3		1	0	-6	-4	-5	-7	-7	-2	-9
	Q	-13	-5	-8	-4	-7	-5	-3	-5	-4	-12	1		-3	-3	-2	1	-14	-13	-2	-7
	N	-9	-7	-5	-9	-8	0	-6	-2	-4	-4	0	-3		-1	2	-2	-11	-8	-6	-3
	K	-14	-8	-6	-2	-9	-4	-6	-3	-7	-9	-6	-3	-1		-4	-4	-14	-12	0	-7
	D	-15	-12	-7	-11	-8	-4	-8	-5	-3	-11	-4	-2	2	-4		2	-14	-15	-10	-3
	E	-14	-9	-5	-7	-6	-4	-5	-6	-2	-8	-5	1	-2	-4	2		-14	-17	-9	-4
G	C	-13	-15	-6	-13	-6	-3	-8	-8	-6	-4	-7	-14	-11	-14	-14	-14		-15	-8	-9
	W	-4	-6	-14	-13	-15	-5	-14	-13	-13	-5	-7	-13	-8	-12	-15	-17	-15		-2	-15
	R	-9	-8	-5	-4	-8	-3	-4	-6	-7	-10	-2	-2	-6	0	-10	-9	-8	-2		-9
	G	-9	-10	-11	-8	-5	-2	-6	-6	-2	-14	-9	-7	-3	-7	-3	-4	-9	-15	-9	

B

	U	C	A	G
U	-1.9	-6.1	-7.8	-9.1
C	-6.1	-1.5	-5.1	-6.6
A	-7.8	-5.1	-3.6	-8.6
G	-9.1	-6.6	-8.6	-9.7

Total average = –6.5

Supplementary Figure 6. Analysis of accepted amino acid replacements in proteins caused by natural selection using PAM matrix. (A) A rearranged PAM30 matrix, where the 20 canonical amino acids are classified into four groups, U, C, A and G. Group U: F, L, I, M and V. Group C: S, P, T and A. Group A: Y, H, Q, N, K, D and E. Group G: C, W, R and G. Scores for pairs of the same amino acids, e.g. (F,F), are omitted. It should be noted that the direction of amino acid replacement is not defined in this matrix. Therefore, the score for (x,y) is identical to that of (y,x), making the table symmetric. See reference 25 for the original data and the calculation method. (B) Average values of the scores for the four groups, U, C, A and G.

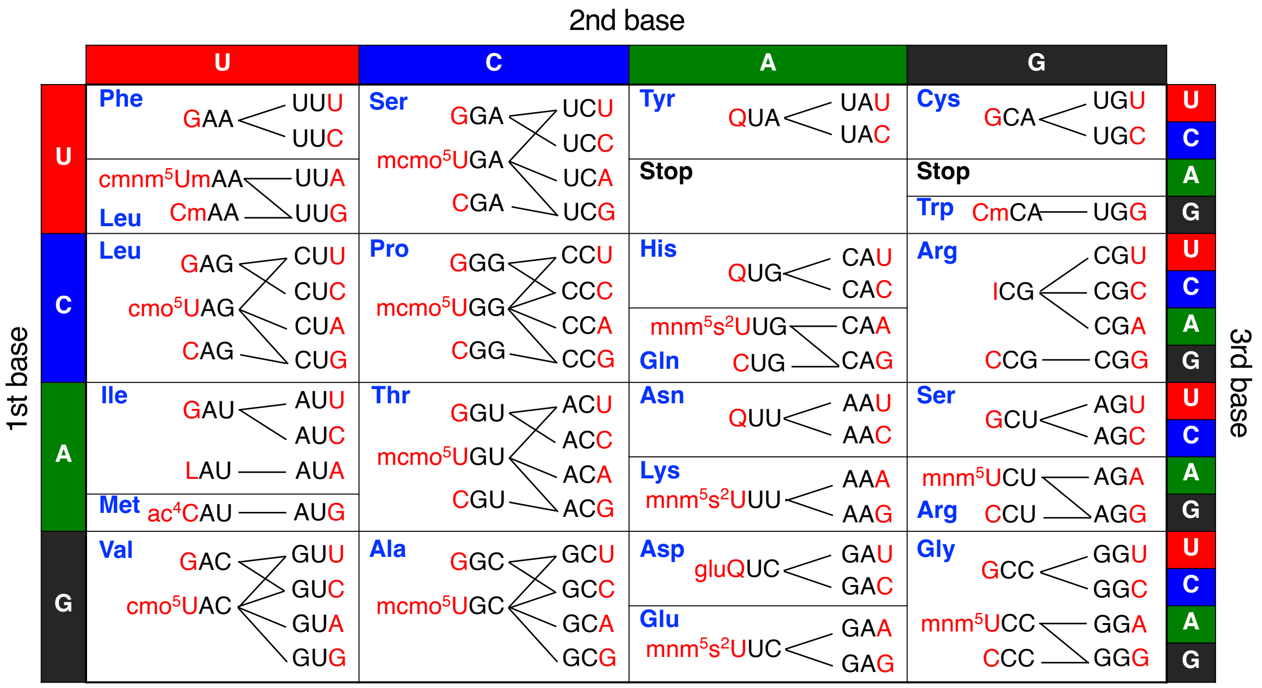

Supplementary Figure 7. Summary of previously reported codon-anticodon interactions and nucleotide modifications in *E. coli.* Anticodon and codon sequences are shown at the left and the right, respectively. The first base of anticodon and the third base of codon are indicated by red. See references 26–34 for details. ac^{4}C: N^4-acetylcytidine, Cm: 2′-O-methylcytidine, cmnm^{5}Um: 5-carboxymethylaminomethyl-2'-O-methyluridine, cmo^{5}U: 5-carboxymethoxyuridine, gluQ: glutamyl-queuosine, I: inosine, mcmo^{5}U: 5-methoxycarbonylmethoxyuridine, mnm^{5}U: 5-methylaminomethyluridine, mnm^{5}s^{2}U: 5-methylaminomethyl-2-thiouridine, Q: queuosine

A

		U					C				A							G			
		F	L	I	M	V	S	P	T	A	Y	H	Q	N	K	D	E	C	W	R	G
UUU	intensity		3	1	3		2		3	2	2			1					2		3
mismatch	1st		±	+	+	+	±	+	+	+	-	+	+	+	+	+	+	-	-	+	+
	2nd		-	-	-	-	+	+	+	+	+	+	+	+	+	+	+	+	+	+	+
	3rd		±	±	+	-	±	±	±	-	-	-	+	-	+	-	+	-	+	±	±
	total		1/2	1/2	2	1	1/2	2/3	2/3	2	1	2	3	2	3	2	3	1	2	2/3	2/3
UUC	intensity		3	3	4	1	3		3	2	3	1		1				1	3		3
mismatch	1st		±	+	+	+	±	+	+	+	-	+	+	+	+	+	+	-	-	+	+
	2nd		-	-	-	-	+	+	+	+	+	+	+	+	+	+	+	+	+	+	+
	3rd		±	±	+	-	±	±	±	-	-	-	+	-	+	-	+	-	+	±	±
	total		1/2	1/2	2	1	1/2	2/3	2/3	2	1	2	3	2	3	2	3	1	2	2/3	2/3
UUA	intensity	4		2	1	3	2		2	1				1							1
mismatch	1st	-		+	+	+	±	+	+	+	-	+	+	+	+	+	+	-	-	+	+
	2nd	-		-	-	-	+	+	+	+	+	+	+	+	+	+	+	+	+	+	+
	3rd	+		±	+	±	±	±	±	±	-	-	±	+	-	+	-	+	+	±	±
	total	1		1/2	2	1/2	1/2/3	2/3	2/3	2/3	1	2	2/3	3	2	3	2	2	2	2/3	2/3
UUG	intensity	2		1	2	1	2		2	1				1					2		1
mismatch	1st	-		+	+	+	±	+	+	+	-	+	+	+	+	+	+	-	-	+	+
	2nd	-		-	-	-	+	+	+	+	+	+	+	+	+	+	+	+	+	+	+
	3rd	+		+	-	±	±	±	±	±	-	-	-	+	-	+	-	+	-	±	±
	total	1		2	1	1/2	1/2/3	2/3	2/3	2/3	1	2	2	3	2	3	2	2	1	2/3	2/3
CUU	intensity	2		2	2	1	1	2	2	1				2				1			1
mismatch	1st	+		+	+	+	+	-	+	+	+	-	-	+	+	+	+	+	+	±	+
	2nd	-		-	-	-	+	+	+	+	+	+	+	+	+	+	+	+	+	+	+
	3rd	-		±	+	-	±	±	±	-	-	-	+	-	+	-	+	-	+	±	±
	total	1		1/2	2	1	2/3	1/2	2/3	2	2	1	2	2	3	2	3	2	3	1/2/3	2/3
CUC	intensity	3		2	2	1	1	3	2	1		1		2				1			1
mismatch	1st	+		+	+	+	+	-	+	+	+	-	-	+	+	+	+	+	+	±	+
	2nd	-		-	-	-	+	+	+	+	+	+	+	+	+	+	+	+	+	+	+
	3rd	-		±	+	-	±	±	±	-	-	-	+	-	+	-	+	-	+	±	±
	total	1		1/2	2	1	2/3	1/2	2/3	2	2	1	2	2	3	2	3	2	3	1/2/3	2/3
CUA	intensity	2		1	1	1	1	2	2	1			2	1				1			1
mismatch	1st	+		+	+	+	+	-	+	+	+	-	-	+	+	+	+	+	+	±	+
	2nd	-		-	-	-	+	+	+	+	+	+	+	+	+	+	+	+	+	+	+
	3rd	+		±	+	±	±	±	±	±	+	+	±	+	-	+	-	+	+	±	±
	total	2		1/2	2	1/2	2/3	1/2	2/3	2/3	3	2	1/2	3	2	3	2	3	3	1/2/3	2/3
CUG	intensity	2		3	2	2	2	3	3	2			1	3				2			2
mismatch	1st	+		+	+	+	+	-	+	+	+	-	-	+	+	+	+	+	+	±	+
	2nd	-		-	-	-	+	+	+	+	+	+	+	+	+	+	+	+	+	+	+
	3rd	+		+	-	±	±	±	±	±	+	+	-	+	-	+	-	+	-	±	±
	total	2		2	1	1/2	2/3	1/2	2/3	2/3	3	2	1	3	2	3	2	3	2	1/2	2/3
AUU	intensity	1	3		3	3			3												
mismatch	1st	+	+		-	+	±	+	-	+	+	+	+	-	-	+	+	+	+	±	+
	2nd	-	-		-	-	+	+	+	+	+	+	+	+	+	+	+	+	+	+	+
	3rd	-	±		+	-	±	±	±	-	-	-	+	-	+	-	+	-	+	±	±
	total	1	1/2		1	1	1/2/3	2/3	1/2	2	2	2	3	1	2	2	3	2	3	1/2/3	2/3
AUC	intensity	1	3		3	4			4					1							
mismatch	1st	+	+		-	+	±	+	-	+	+	+	+	-	-	+	+	+	+	±	+
	2nd	-	-		-	-	+	+	+	+	+	+	+	+	+	+	+	+	+	+	+
	3rd	-	±		+	-	±	±	±	-	-	-	+	-	+	-	+	-	+	±	±
	total	1	1/2		1	1	1/2/3	2/3	1/2	2	2	2	3	1	2	2	3	2	3	1/2/3	2/3
AUA	intensity		1		5	2			2												
mismatch	1st	+	+		-	+	±	+	-	+	+	+	+	-	-	+	+	+	+	±	+
	2nd	-	-		-	-	+	+	+	+	+	+	+	+	+	+	+	+	+	+	+
	3rd	+	±		+	±	±	±	±	±	+	+	±	+	-	+	-	+	+	±	±
	total	2	1/2		1	1/2	2/3	2/3	1/2	2/3	3	3	2/3	2	1	3	2	3	3	1/2/3	2/3
AUG	intensity		1	3					4												
mismatch	1st	+	+	-		+	±	+	-	+	+	+	+	-	-	+	+	+	+	±	+
	2nd	-	-	-		-	+	+	+	+	+	+	+	+	+	+	+	+	+	+	+
	3rd	+	±	+		±	±	±	±	±	+	+	-	+	-	+	-	-	-	±	±
	total	2	1/2	1		1/2	2/3	2/3	1/2	2/3	3	3	2	2	1	3	2	2	2	1/2/3	2/3
GUU	intensity		3	5	2		1	3	4	4				2		1		4			2
mismatch	1st	+	+	+	+		+	+	+	-	+	+	+	+	+	-	-	+	+	+	-
	2nd	-	-	-	-		+	+	+	+	+	+	+	+	+	+	+	+	+	+	+
	3rd	-	±	±	+		±	±	±	-	-	-	+	-	+	-	+	-	+	±	±
	total	1	1/2	1/2	2		2/3	2/3	2/3	1	2	2	3	2	3	1	2	2	3	2/3	1/2
GUC	intensity		3	5	2		1	3	4	4				2		1		4			1
mismatch	1st	+	+	+	+		+	+	+	-	+	+	+	+	+	-	-	+	+	+	-
	2nd	-	-	-	-		+	+	+	+	+	+	+	+	+	+	+	+	+	+	+
	3rd	-	±	±	+		±	±	±	-	-	-	+	-	+	-	+	-	+	±	±
	total	1	1/2	1/2	2		2/3	2/3	2/3	1	2	2	3	2	3	1	2	2	3	2/3	1/2
GUA	intensity		3	5	2		1	3	4	4				2		1		4			1
mismatch	1st	+	+	+	+		+	+	+	-	+	+	+	+	+	-	-	+	+	+	-
	2nd	-	-	-	-		+	+	+	+	+	+	+	+	+	+	+	+	+	+	+
	3rd	+	±	±	+		±	±	±	±	+	+	±	+	-	+	-	+	+	±	±
	total	2	1/2	1/2	2		2/3	2/3	2/3	1/2	3	3	2/3	3	2	2	1	3	3	2/3	1/2
GUG	intensity		3	4	5		1	3	3	2				2		1		4			2
mismatch	1st	+	+	+	+		+	+	+	-	+	+	+	+	+	-	-	+	+	+	-
	2nd	-	-	-	-		+	+	+	+	+	+	+	+	+	+	+	+	+	+	+
	3rd	+	±	+	-		±	±	±	±	+	+	-	+	-	+	-	+	-	±	±
	total	2	1/2	2	1		2/3	2/3	2/3	1/2	3	3	2	3	2	2	1	3	2	2/3	1/2

Supplementary Figure 8. Number of mismatches between codon and anticodon for 1,159 codon-amino acid combinations. (A) NUN codons, (B) NCN codons, (C) NAN codons and (D) NGN codons. Match and mismatch at each position are indicated by – and +, respectively. ± indicates there are multiple isoacceptor tRNAs that form both matched and mismatched base pairs with designated codon. Intensity of peptide bearing misincorporation of designated amino acid is shown at the top. Number of mismatches is shown at the bottom.

B

		U					C				A							G			
		F	L	I	M	V	S	P	T	A	Y	H	Q	N	K	D	E	C	W	R	G
UCU	intensity	1	1	1				2	2	1											1
mismatch	1st	-	±	+	+	+		+	+	+	-	+	+	+	+	+	+	-	-	+	+
	2nd	+	+	+	+	+		-	-	-	+	+	+	+	+	+	+	+	+	+	+
	3rd	-	±	±	+	-		±	±	-	-	-	+	-	+	-	+	-	+	±	±
	total	1	2/3	2/3	3	2		1/2	1/2	2	1	2	3	2	3	2	3	1	2	2/3	2/3
UCC	intensity	2		1				2	2	1											1
mismatch	1st	-	±	+	+	+		+	+	+	-	+	+	+	+	+	+	-	-	+	+
	2nd	+	+	+	+	+		-	-	-	+	+	+	+	+	+	+	+	+	+	+
	3rd	-	±	±	+	-		±	±	-	-	-	+	-	+	-	+	-	+	±	±
	total	1	2/3	2/3	3	2		1/2	1/2	2	1	2	3	2	3	2	3	1	2	2/3	2/3
UCA	intensity	1	1	1				2	1	1											1
mismatch	1st	-	±	+	+	+		+	+	+	-	+	+	+	+	+	+	-	-	+	+
	2nd	+	+	+	+	+		-	-	-	+	+	+	+	+	+	+	+	+	+	+
	3rd	+	±	±	+	±		±	±	±	+	+	±	+	-	+	-	+	+	±	±
	total	2	1/2/3	2/3	3	2/3		1/2	1/2	1/2	2	3	2/3	3	2	3	2	2	2	2/3	2/3
UCG	intensity		2					1	1	1											1
mismatch	1st	-	±	+	+	+		+	+	+	-	+	+	+	+	+	+	-	-	+	+
	2nd	+	+	+	+	+		-	-	-	+	+	+	+	+	+	+	+	+	+	+
	3rd	+	±	±	-	±		±	±	±	+	+	-	+	-	+	-	+	-	±	±
	total	2	1/2/3	2/3	2	2/3		1/2	1/2	1/2	2	3	2	3	2	3	2	2	1	2/3	2/3
CCU	intensity		3	4	1	3	3		3	3	2		2	1						2	2
mismatch	1st	+	±	+	+	+	+		+	+	+	-	-	+	+	+	+	+	+	±	+
	2nd	+	+	+	+	+	±		-	-	+	+	+	+	+	+	+	+	+	+	+
	3rd	-	±	±	+	-	±		±	-	-	-	+	-	+	-	+	-	+	±	±
	total	2	1/2/3	2/3	3	2	1/2		1/2	1	2	1	2	2	3	2	3	2	3	1/2/3	2/3
CCC	intensity		3	3	1	3	2		3	2											1
mismatch	1st	+	±	+	+	+	+		+	+	+	-	-	+	+	+	+	+	+	±	+
	2nd	+	+	+	+	+	±		-	-	+	+	+	+	+	+	+	+	+	+	+
	3rd	-	±	±	+	-	±		±	-	-	-	+	-	+	-	+	-	+	±	±
	total	2	1/2/3	2/3	3	2	1/2		1/2	1	2	1	2	2	3	2	3	2	3	1/2/3	2/3
CCA	intensity		2	3	1	3	3		4	3			2	1							2
mismatch	1st	+	±	+	+	+	+		+	+	+	-	-	+	+	+	+	+	+	±	+
	2nd	+	+	+	+	+	±		-	-	+	+	+	+	+	+	+	+	+	+	+
	3rd	+	±	±	+	±	±		±	±	+	+	-	+	-	+	-	+	+	±	±
	total	3	1/2	2/3	3	2/3	1/2/3		1/2	1/2	3	2	1	3	2	3	2	3	3	1/2/3	2/3
CCG	intensity		3	3	1	3	3		3	3		1	1	1							2
mismatch	1st	+	±	+	+	+	+		+	+	+	-	-	+	+	+	+	+	+	±	+
	2nd	+	+	+	+	+	±		-	-	+	+	+	+	+	+	+	+	+	+	+
	3rd	+	±	+	-	±	±		±	±	+	+	-	+	-	+	-	+	-	±	±
	total	3	1/2	3	2	2/3	1/2/3		1/2	1/2	3	2	1	3	2	3	2	3	2	1/2	2/3
ACU	intensity	1	1	1		1	5			2				1		1		3			1
mismatch	1st	+	+	-	-	+	±	+		+	+	+	+	-	-	+	+	+	+	±	+
	2nd	+	+	+	+	+	±	-		-	+	+	+	+	+	+	+	+	+	+	+
	3rd	-	±	±	+	-	±	±		-	-	-	+	-	+	-	+	-	+	±	±
	total	2	2/3	1/2	2	2	1/2	1/2		1	2	2	3	1	2	2	3	2	3	2/3	2/3
ACC	intensity		1	1	1	2	5			2				1		1		3			1
mismatch	1st	+	+	-	-	+	±	+		+	+	+	+	-	-	+	+	+	+	±	+
	2nd	+	+	+	+	+	±	-		-	+	+	+	+	+	+	+	+	+	+	+
	3rd	-	±	±	+	-	±	±		-	-	-	+	-	+	-	+	-	+	±	±
	total	2	2/3	1/2	2	2	1/2	1/2		1	2	2	3	1	2	2	3	2	3	2/3	2/3
ACA	intensity						4			1								1			
mismatch	1st	+	+	-	-	+	±	+		+	+	+	+	-	-	+	+	+	+	±	+
	2nd	+	+	+	+	+	±	-		-	+	+	+	+	+	+	+	+	+	+	+
	3rd	+	±	±	+	±	±	±		±	+	+	±	+	-	+	-	-	+	±	±
	total	3	2/3	1/2	2	2/3	1/2	1/2		1/2	3	3	2/3	2	1	3	2	2	3	1/2/3	2/3
ACG	intensity				1	1	4			2								1			
mismatch	1st	+	+	-	-	+	±	+		+	+	+	+	-	-	+	+	+	+	±	+
	2nd	+	+	+	+	+	±	-		-	+	+	+	+	+	+	+	+	+	+	+
	3rd	+	±	+	-	±	±	±		±	+	+	-	+	-	+	-	-	-	±	±
	total	3	2/3	2	1	2/3	1/2	1/2		1/2	3	3	2	2	1	3	2	2	2	1/2/3	2/3
GCU	intensity				1	2	5	3	3									1			2
mismatch	1st	+	+	+	+	-	+	+	+		+	+	+	+	+	-	-	+	+	+	-
	2nd	+	+	+	+	+	±	-	-		+	+	+	+	+	+	+	+	+	+	+
	3rd	-	±	±	+	-	±	±	±		-	-	+	-	+	-	+	-	+	±	±
	total	2	2/3	2/3	3	1	1/2	1/2	1/2		2	2	3	2	3	1	2	2	3	2/3	1/2
GCC	intensity				1	1	5	2	3									2			5
mismatch	1st	+	+	+	+	-	+	+	+		+	+	+	+	+	-	-	+	+	+	-
	2nd	+	+	+	+	+	±	-	-		+	+	+	+	+	+	+	+	+	+	+
	3rd	-	±	±	+	-	±	±	±		-	-	+	-	+	-	+	-	+	±	±
	total	2	2/3	2/3	3	1	1/2	1/2	1/2		2	2	3	2	3	1	2	2	3	2/3	1/2
GCA	intensity				1	1	5	2	3									1			5
mismatch	1st	+	+	+	+	-	+	+	+		+	+	+	+	+	-	-	+	+	+	-
	2nd	+	+	+	+	+	±	-	-		+	+	+	+	+	+	+	+	+	+	+
	3rd	+	±	±	+	±	±	±	±		+	+	±	+	-	+	-	+	+	±	±
	total	3	2/3	2/3	3	1/2	1/2/3	1/2	1/2		3	3	2/3	3	2	2	1	3	3	2/3	1/2
GCG	intensity				1	1	5	2	2									1			4
mismatch	1st	+	+	+	+	-	+	+	+		+	+	+	+	+	-	-	+	+	+	-
	2nd	+	+	+	+	+	±	-	-		+	+	+	+	+	+	+	+	+	+	+
	3rd	+	±	+	-	±	±	±	±		+	+	-	+	-	+	-	+	-	±	±
	total	3	2/3	3	2	1/2	1/2/3	1/2	1/2		3	3	2	3	2	2	1	3	2	2/3	1/2

Supplementary Figure 8 continued.

C

		U					C				A							G			
		F	L	I	M	V	S	P	T	A	Y	H	Q	N	K	D	E	C	W	R	G
**UAU**	intensity				1							1		3							
mismatch	1st	-	±	+	+	+	±	+	+	+		+	+	+	+	+	+	-	-	+	+
	2nd	+	+	+	+	+	+	+	+	+		-	-	-	-	-	-	+	+	+	+
	3rd	-	±	±	+	-	±	±	±	-		-	+	-	+	-	+	-	+	±	±
	total	1	2/3	2/3	3	2	1/2	2/3	2/3	2		1	2	1	2	1	2	1	2	2/3	2/3
**UAC**	intensity				1							2		3				1			
mismatch	1st	-	±	+	+	+	±	+	+	+		+	+	+	+	+	+	-	-	+	+
	2nd	+	+	+	+	+	+	+	+	+		-	-	-	-	-	-	+	+	+	+
	3rd	-	±	±	+	-	±	±	±	-		-	+	-	+	-	+	-	+	±	±
	total	1	2/3	2/3	3	2	1/2	2/3	2/3	2		1	2	1	2	1	2	1	2	2/3	2/3
**CAU**	intensity	1			1		1			2	1		4	1					2		1
mismatch	1st	+	±	+	+	+	+	-	+	+	+		-	+	+	+	+	+	+	±	+
	2nd	+	+	+	+	+	+	+	+	+	-		-	-	-	-	-	+	+	+	+
	3rd	-	±	±	+	-	±	±	±	-	-		+	-	+	-	+	-	+	±	±
	total	2	1/2/3	2/3	3	2	2/3	1/2	2/3	2	1		1	1	2	1	2	2	3	1/2/3	2/3
**CAC**	intensity	1			2					2	1		3	3					2		1
mismatch	1st	+	±	+	+	+	+	-	+	+	+		-	+	+	+	+	+	+	±	+
	2nd	+	+	+	+	+	+	+	+	+	-		-	-	-	-	-	+	+	+	+
	3rd	-	±	±	+	-	±	±	±	-	-		+	-	+	-	+	-	+	±	±
	total	2	1/2/3	2/3	3	2	2/3	1/2	2/3	2	1		1	1	2	1	2	2	3	1/2/3	2/3
**CAA**	intensity						1			3		1									2
mismatch	1st	+	±	+	+	+	+	-	+	+	+	-		+	+	+	+	+	+	±	+
	2nd	+	+	+	+	+	+	+	+	+	-	-		-	-	-	-	+	+	+	+
	3rd	+	±	±	+	±	±	±	±	±	+	+		+	-	+	-	+	+	±	±
	total	3	1/2	2/3	3	2/3	2/3	1/2	2/3	2/3	2	1		2	1	2	1	3	3	1/2/3	2/3
**CAG**	intensity						1			3											2
mismatch	1st	+	±	+	+	+	+	-	+	+	+	-		+	+	+	+	+	+	±	+
	2nd	+	+	+	+	+	+	+	+	+	-	-		-	-	-	-	+	+	+	+
	3rd	+	±	+	-	±	±	±	±	±	+	+		+	-	+	-	+	-	±	±
	total	3	1/2	3	2	2/3	2/3	1/2	2/3	2/3	2	1		2	1	2	1	3	2	1/2	2/3
**AAU**	intensity						4			1					2	2					1
mismatch	1st	+	+	-	-	+	±	+	-	+	+	+	+		-	+	+	+	+	±	+
	2nd	+	+	+	+	+	+	+	+	+	-	-	-		-	-	-	+	+	+	+
	3rd	-	±	±	+	-	±	±	±	-	-	-	+		+	-	+	-	+	±	±
	total	2	2/3	1/2	2	2	1/2/3	2/3	1/2	2	1	1	2		1	1	2	2	3	2/3	2/3
**AAC**	intensity						4			1					2	2					1
mismatch	1st	+	+	-	-	+	±	+	-	+	+	+	+		-	+	+	+	+	±	+
	2nd	+	+	+	+	+	+	+	+	+	-	-	-		-	-	-	+	+	+	+
	3rd	-	±	±	+	-	±	±	±	-	-	-	+		+	-	+	-	+	±	±
	total	2	2/3	1/2	2	2	1/2/3	2/3	1/2	2	1	1	2		1	1	2	2	3	2/3	2/3
**AAA**	intensity								1	1											
mismatch	1st	+	+	-	-	+	±	+	-	+	+	+	+	-		+	+	+	+	±	+
	2nd	+	+	+	+	+	+	+	+	+	-	-	-	-		-	-	+	+	+	+
	3rd	+	±	±	+	±	±	±	±	±	+	+	±	+		+	-	+	+	±	±
	total	3	2/3	1/2	2	2/3	2/3	2/3	1/2	2/3	2	2	1/2	1		2	1	3	3	1/2/3	2/3
**AAG**	intensity	1			2				2	2								1			
mismatch	1st	+	+	-	-	+	±	+	-	+	+	+	+	-		+	+	+	+	±	+
	2nd	+	+	+	+	+	+	+	+	+	-	-	-	-		-	-	+	+	+	+
	3rd	+	±	+	-	±	±	±	±	±	+	+	-	+		+	-	+	-	±	±
	total	3	2/3	2	1	2/3	2/3	2/3	1/2	2/3	2	2	1	1		2	1	3	2	1/2/3	2/3
**GAU**	intensity																2				
mismatch	1st	+	+	+	+	-	+	+	+	-	+	+	+	+	+		-	+	+	+	-
	2nd	+	+	+	+	+	+	+	+	+	-	-	-	-	-		-	+	+	+	+
	3rd	-	±	±	+	-	±	±	±	-	-	-	+	-	+		+	-	+	±	±
	total	2	2/3	2/3	3	1	2/3	2/3	2/3	1	1	1	2	1	2		1	2	3	2/3	1/2
**GAC**	intensity																1	1			
mismatch	1st	+	+	+	+	-	+	+	+	-	+	+	+	+	+		-	+	+	+	-
	2nd	+	+	+	+	+	+	+	+	+	-	-	-	-	-		-	+	+	+	+
	3rd	-	±	±	+	-	±	±	±	-	-	-	+	-	+		+	-	+	±	±
	total	2	2/3	2/3	3	1	2/3	2/3	2/3	1	1	1	2	1	2		1	2	3	2/3	1/2
**GAA**	intensity						1			2			1		2						3
mismatch	1st	+	+	+	+	-	+	+	+	-	+	+	+	+	+	-		+	+	+	-
	2nd	+	+	+	+	+	+	+	+	+	-	-	-	-	-	-		+	+	+	+
	3rd	+	±	±	+	±	±	±	±	±	+	+	±	+	-	+		+	+	±	±
	total	3	2/3	2/3	3	1/2	2/3	2/3	2/3	1/2	2	2	1/2	2	1	1		3	3	2/3	1/2
**GAG**	intensity									1											2
mismatch	1st	+	+	+	+	-	+	+	+	-	+	+	+	+	+	-		+	+	+	-
	2nd	+	+	+	+	+	+	+	+	+	-	-	-	-	-	-		+	+	+	+
	3rd	+	±	+	-	±	±	±	±	±	+	+	-	+	-	+		+	-	±	±
	total	3	2/3	3	2	1/2	2/3	2/3	2/3	1/2	2	2	1	2	1	1		3	2	2/3	1/2

Supplementary Figure 8 continued.

D

		U					C				A							G			
		F	L	I	M	V	S	P	T	A	Y	H	Q	N	K	D	E	C	W	R	G
UGU	intensity						1				2										
mismatch	1st	-	±	+	+	+	±	+	+	+	-	+	+	+	+	+	+		-	+	+
	2nd	+	+	+	+	+	±	+	+	+	+	+	+	+	+	+	+		-	-	-
	3rd	-	±	±	+	-	±	±	±	-	-	-	+	-	+	-	+		+	±	±
	total	1	2/3	2/3	3	2	1/2	2/3	2/3	2	1	2	3	2	3	2	3		1	1/2	1/2
UGC	intensity						2				4								1	1	
mismatch	1st	-	±	+	+	+	±	+	+	+	-	+	+	+	+	+	+		-	+	+
	2nd	+	+	+	+	+	±	+	+	+	+	+	+	+	+	+	+		-	-	-
	3rd	-	±	±	+	-	±	±	±	-	-	-	+	-	+	-	+		+	±	±
	total	1	2/3	2/3	3	2	1/2	2/3	2/3	2	1	2	3	2	3	2	3		1	1/2	1/2
UGG	intensity	3																			1
mismatch	1st	-	±	+	+	+	±	+	+	+	-	+	+	+	+	+	+	-		+	+
	2nd	+	+	+	+	+	±	+	+	+	+	+	+	+	+	+	+	-		-	-
	3rd	+	±	+	-	±	±	±	±	±	+	+	-	+	-	+	-	+		±	±
	total	2	1/2/3	3	2	2/3	1/2	2/3	2/3	2/3	2	3	2	3	2	3	2	1		1/2	1/2
CGU	intensity	1										1									
mismatch	1st	+	±	+	+	+	+	-	+	+	+	-	-	+	+	+	+	+	+		+
	2nd	+	+	+	+	+	±	+	+	+	+	+	+	+	+	+	+	-	-		-
	3rd	-	±	±	+	-	±	±	±	-	-	-	+	-	+	-	+	-	+		±
	total	2	1/2/3	2/3	3	2	1/2/3	1/2	2/3	2	2	1	2	2	3	2	3	1	2		1/2
CGC	intensity											2						3			
mismatch	1st	+	±	+	+	+	+	-	+	+	+	-	-	+	+	+	+	+	+		+
	2nd	+	+	+	+	+	±	+	+	+	+	+	+	+	+	+	+	-	-		-
	3rd	-	±	±	+	-	±	±	±	-	-	-	+	-	+	-	+	-	+		±
	total	2	1/2/3	2/3	3	2	1/2/3	1/2	2/3	2	2	1	2	2	3	2	3	1	2		1/2
CGA	intensity												5	4							
mismatch	1st	+	±	+	+	+	+	-	+	+	+	-	-	+	+	+	+	+	+		+
	2nd	+	+	+	+	+	±	+	+	+	+	+	+	+	+	+	+	-	-		-
	3rd	+	±	±	+	±	±	±	±	±	+	+	±	+	-	+	-	+	+		±
	total	3	1/2	2/3	3	2/3	2/3	1/2	2/3	2/3	3	2	1/2	3	2	3	2	2	2		1/2
CGG	intensity												5								1
mismatch	1st	+	±	+	+	+	+	-	+	+	+	-	-	+	+	+	+	+	+		+
	2nd	+	+	+	+	+	±	+	+	+	+	+	+	+	+	+	+	-	-		-
	3rd	+	±	+	-	±	±	±	±	±	+	+	-	+	-	+	-	+	-		±
	total	3	1/2	3	2	2/3	2/3	1/2	2/3	2/3	3	2	1	3	2	3	2	2	1		1/2
AGU	intensity								1					3							
mismatch	1st	+	+	-	-	+		+	-	+	+	+	+	-	-	+	+	+	+	±	+
	2nd	+	+	+	+	+		+	+	+	+	+	+	+	+	+	+	-	-	-	-
	3rd	-	±	±	+	-		±	±	-	-	-	+	-	+	-	+	-	+	±	±
	total	2	2/3	1/2	2	2		2/3	1/2	2	2	2	2	1	2	2	3	1	2	1/2	1/2
AGC	intensity								1					4							1
mismatch	1st	+	+	-	-	+		+	-	+	+	+	+	-	-	+	+	+	+	±	+
	2nd	+	+	+	+	+		+	+	+	+	+	+	+	+	+	+	-	-	-	-
	3rd	-	±	±	+	-		±	±	-	-	-	+	-	+	-	+	-	+	±	±
	total	2	2/3	1/2	2	2		2/3	1/2	2	2	2	2	1	2	2	3	1	2	1/2	1/2
AGA	intensity						3								2						
mismatch	1st	+	+	-	-	+	±	+	-	+	+	+	+	-	-	+	+	+	+		+
	2nd	+	+	+	+	+	±	+	+	+	+	+	+	+	+	+	+	-	-		-
	3rd	+	±	±	+	±	±	±	±	±	+	+	±	+	-	+	-	+	+		±
	total	3	2/3	1/2	2	2/3	1/2/3	2/3	1/2	2/3	3	3	2/3	2	1	3	2	2	2		1/2
AGG	intensity						1														1
mismatch	1st	+	+	-	-	+	±	+	-	+	+	+	+	-	-	+	+	+	+		+
	2nd	+	+	+	+	+	±	+	+	+	+	+	+	+	+	+	+	-	-		-
	3rd	+	±	+	-	±	±	±	±	±	+	+	-	+	-	+	-	+	-		±
	total	3	2/3	2	1	2/3	1/2/3	2/3	1/2	2/3	3	3	2	2	1	3	2	2	1		1/2
GGU	intensity						2			1											
mismatch	1st	+	+	+	+	-	+	+	+	-	+	+	+	+	+	-	-	+	+	+	
	2nd	+	+	+	+	+	±	+	+	+	+	+	+	+	+	+	+	-	-	-	
	3rd	-	±	±	+	-	±	±	±	-	-	-	+	-	+	-	+	-	+	±	
	total	2	2/3	2/3	3	1	1/2/3	2/3	2/3	1	2	2	3	2	3	1	2	1	2	1/2	
GGC	intensity						4			1						4					
mismatch	1st	+	+	+	+	-	+	+	+	-	+	+	+	+	+	-	-	+	+	+	
	2nd	+	+	+	+	+	±	+	+	+	+	+	+	+	+	+	+	-	-	-	
	3rd	-	±	±	+	-	±	±	±	-	-	-	+	-	+	-	+	-	+	±	
	total	2	2/3	2/3	3	1	1/2/3	2/3	2/3	1	2	2	3	2	3	1	2	1	2	1/2	
GGA	intensity									1							3				
mismatch	1st	+	+	+	+	-	+	+	+	-	+	+	+	+	+	-	-	+	+	+	
	2nd	+	+	+	+	+	±	+	+	+	+	+	+	+	+	+	+	-	-	-	
	3rd	+	±	±	+	±	±	±	±	±	+	+	±	+	-	+	-	+	+	±	
	total	3	2/3	2/3	3	1/2	2/3	2/3	2/3	1/2	3	3	2/3	3	2	2	1	2	2	1/2	
GGG	intensity									1		1								1	
mismatch	1st	+	+	+	+	-	+	+	+	-	+	+	+	+	+	-	-	+	+	+	
	2nd	+	+	+	+	+	±	+	+	+	+	+	+	+	+	+	+	-	-	-	
	3rd	+	±	+	-	±	±	±	±	±	+	+	-	+	-	+	-	+	-	±	
	total	3	2/3	3	2	1/2	2/3	2/3	2/3	1/2	3	3	2	3	2	2	1	2	1	1/2	

Supplementary Figure 8 continued.

A

P1-Xaa	*m/z* [M+4H]$^{4+}$	retention time (min)
Gly	526.490	5.271
Ala	529.994	5.444
Ser	533.993	5.271
Pro	536.498	5.602
Val	537.002	5.885
Thr	537.496	5.412
Cys	537.987	5.607
Ile	540.505	6.190
Leu	540.505	6.274
Asn	540.745	5.218
Asp	540.991	5.328
Gln	544.249	5.239
Lys	544.258	4.850
Glu	544.495	5.328
Met	544.995	5.943
His	546.499	4.898
Phe	549.002	6.405
Arg	551.260	4.908
Tyr	553.000	5.749
Trp	558.754	6.416

B

P2-Xaa	*m/z* [M+4H]$^{4+}$	retention time (min)
Thr	545.007	6.106
Ile	548.016	6.815
Leu	548.016	6.899

C

P3-Xaa	*m/z* [M+4H]$^{4+}$	retention time (min)
Cys	521.992	8.401
Asn	524.750	7.870
Met	529.000	8.737
His	530.504	7.377

D

P4-Xaa	*m/z* [M+4H]$^{4+}$	retention time (min)
Ala	565.001	5.670
Thr	572.504	5.644
Cys	572.994	5.833
Met	580.002	6.164
Phe	584.009	6.600

E

P5-Xaa	*m/z* [M+4H]$^{4+}$	retention time (min)
Cys	502.993	5.271
Glu	509.501	5.030

Supplementary Table 2. *m/z* and retention time of authentic peptides in LC/MS analysis. Values for P1-Xaa (A), P2-Xaa (B), P3-Xaa (C), P4-Xaa (D) and P5-Xaa (E).

	codon	mRNA/ peptide	isotope label
NUN	UUU	mR1/P1	[$^{13}C_6$]-Ile
	UUC	mR1/P1	[$^{13}C_6$]-Ile
	UUA	mR1/P1	[$^{13}C_6$]-Ile
	UUG	mR1/P1	[$^{13}C_6$]-Ile
	CUU	mR1/P1	[$^{13}C_6$]-Ile
	CUC	mR1/P1	[$^{13}C_6$]-Ile
	CUA	mR1/P1	[$^{13}C_6$]-Ile
	CUG	mR1/P1	[$^{13}C_6$]-Ile
	AUU	mR1/P1	[D_{10}]-Leu
	AUC	mR1/P1	[D_{10}]-Leu
	AUA	mR1/P1	[D_{10}]-Leu
	AUG	mR2/P2	[$^{13}C_6$]-Ile
	GUU	mR1/P1	[D_{10}]-Leu
	GUC	mR1/P1	[D_{10}]-Leu
	GUA	mR1/P1	[D_{10}]-Leu
	GUG	mR1/P1	[D_{10}]-Leu

	codon	mRNA/ peptide	isotope label
NCN	UCU	mR1/P1	[$^{13}C_6$]-Ile
	UCC	mR1/P1	[$^{13}C_6$]-Ile
	UCA	mR1/P1	[$^{13}C_6$]-Ile
	UCG	mR1/P1	[$^{13}C_6$]-Ile
	CCU	mR1/P1	[$^{13}C_6$]-Ile
	CCC	mR1/P1	[$^{13}C_6$]-Ile
	CCA	mR1/P1	[$^{13}C_6$]-Ile
	CCG	mR1/P1	[$^{13}C_6$]-Ile
	ACU	mR1/P1	[$^{13}C_6$]-Ile
	ACC	mR1/P1	[$^{13}C_6$]-Ile
	ACA	mR1/P1	N.A.
	ACG	mR1/P1	N.A.
	GCU	mR1/P1	N.A.
	GCC	mR1/P1	N.A.
	GCA	mR1/P1	N.A.
	GCG	mR1/P1	N.A.

	codon	mRNA/ peptide	isotope label
NAN	UAU	mR3/P3	N.A.
	UAC	mR3/P3	N.A.
	CAU	mR1/P1	N.A.
	CAC	mR1/P1	N.A.
	CAA	mR1/P1	N.A.
	CAG	mR1/P1	N.A.
	AAU	mR1/P1	N.A.
	AAC	mR1/P1	N.A.
	AAA	mR4/P4	N.A.
	AAG	mR4/P4	N.A.
	GAU	mR5/P5	N.A.
	GAC	mR5/P5	N.A.
	GAA	mR1/P1	N.A.
	GAG	mR1/P1	N.A.

	codon	mRNA/ peptide	isotope label
NGN	UGU	mR1/P1	N.A.
	UGC	mR1/P1	N.A.
	UGG	mR1/P1	N.A.
	CGU	mR1/P1	N.A.
	CGC	mR1/P1	N.A.
	CGA	mR1/P1	N.A.
	CGG	mR1/P1	N.A.
	AGU	mR1/P1	N.A.
	AGC	mR1/P1	N.A.
	AGA	mR1/P1	N.A.
	AGG	mR1/P1	N.A.
	GGU	mR1/P1	N.A.
	GGC	mR1/P1	N.A.
	GGA	mR1/P1	N.A.
	GGG	mR1/P1	N.A.

Supplementary Table 3. List of mRNA, peptide and isotope labeled amino acid used for evaluation of misdecoding at 61 sense codons.

misincorporating		misincorporated			
amino acid	tRNA(anticodon)	mRNA(codon)		corresponding amino acids	
A	GGC,cmo5UGC	UUU(F), UUC(F), UUA(L), UUG(L), CUU(L), CUC(L), CUA(L), CUG(L), GUU(V), GUC(V), GUA(V), GUG(V), UCU(S), UCC(S), UCA(S), UCG(S), CCU(P), CCC(P), CCA(P), CCG(P), ACU(T), ACC(T), ACA(T), ACG(T), CAU(H), CAC(H), CAA(Q), CAG(Q), AAU(N), AAC(N), AAA(K), AAG(K), GAA(E), GAG(E), GGU(G), GGC(G), GGA(G), GGG(G)	38	F,L,V,S,P,T,H,Q,N,K,E,G	12
C	GCA	UUC(F), CUU(L), CUC(L), CUA(L), CUG(L), GUU(V), GUC(V), GUA(V), GUG(V), ACU(T), ACC(T), ACA(T), ACG(T), GCU(A), GCC(A), GCA(A), GCG(A), UAC(Y), AAG(K), GAC(D), CGC(R)	21	F,L,V,T,A,Y,K,D,R	9
D	gluQUC	GUU(V), GUC(V), GUA(V), GUG(V), ACU(T), ACC(T), AAU(N), AAC(N), GGC(G)	9	V,T,N,G	4
E	mnm5s2UUC	GAU(D),GAC(D), GGA(G)	3	D,G	2
F	GAA	UUA(L), UUG(L), CUU(L), CUC(L), CUA(L), CUG(L), AUU(I), AUC(I), UCU(S), UCC(S), UCA(S), ACU(T), CAU(H), CAC(H), AAG(K), UGG(W), CGU(R)	17	L,I,S,T,H,K,W,R	8
G	GCC,CCC,mnm5UCC	UUU(F), UUC(F), UUA(L), UUG(L), CUU(L), CUC(L), CUA(L), CUG(L), GUU(V), GUC(V), GUA(V), GUG(V), UCU(S), UCC(S), UCA(S), UCG(S), CCU(P), CCC(P), CCA(P), CCG(P), ACU(T), ACC(T), GCU(A), GCC(A), GCA(A), GCG(A), CAU(H), CAC(H), CAA(Q), CAG(Q), AAU(N), AAC(N), GAA(E), GAG(E), UGG(W), CGG(R), AGC(S), AGG(R)	38	F,L,V,S,P,T,A,H,Q,N,E,W,R	13
H	QUG	UUC(F), CUC(L), CCG(P), UAU(Y), UAC(Y), CAA(Q), CGU(R), CGC(R), GGG(G)	9	F,L,P,Y,Q,R,G	7
I	GAU,k2CAU	UUU(F), UUC(F), UUA(L), UUG(L), CUU(L), CUC(L), CUA(L), CUG(L), AUG(M), GUU(V), GUC(V), GUA(V), GUG(V), UCU(S), UCC(S), UCA(S), CCU(P), CCC(P), CCA(P), CCG(P), ACU(T), ACC(T)	22	F,L,M,V,S,P,T	7
K	mnm5s2UUU	AAU(N), AAC(N), GAA(E), AGA(R)	4	N,E,R	3
L	cmnm5UmAA,CmAA,GAG,CAG,cmo5UAG	UUU(F), UUC(F), AUU(I), AUC(I), AUA(I), AUG(M), GUU(V), GUC(V), GUA(V), GUG(V), UCU(S), UCA(S), UCG(S), CCU(P), CCC(P), CCA(P), CCG(P), ACU(T), ACC(T)	19	F,I,M,V,S,P,T	7
M	ac4CAU	UUU(F), UUC(F), UUA(L), UUG(L), CUU(L), CUC(L), CUA(L), CUG(L), AUU(I), AUC(I), AUA(I), GUU(V), GUC(V), GUA(V), GUG(V), CCU(P), CCC(P), CCA(P), CCG(P), ACC(T), ACG(T), GCU(A), GCC(A), GCA(A), GCG(A), UAU(Y), UAC(Y), CAU(H), CAC(H), AAG(K)	30	F,L,I,V,P,T,A,Y,H,K	10
N	QUU	UUU(F), UUC(F), UUA(L), UUG(L), CUU(L), CUC(L), CUA(L), CUG(L), AUCU(I), GUU(V), GUC(V), GUA(V), GUG(V), CCU(P), CCA(P), CCG(P), ACU(T), ACC(T), UAU(Y), UAC(Y), CAU(H), CAC(H), CGA(R), AGU(S), AGC(S)	25	F,L,I,V,P,T,Y,H,R,S	10
P	GGG,CGG,cmo5UGG	CUU(L), CUC(L), CUA(L), CUG(L), GUU(V), GUC(V), GUA(V), GUG(V), UCU(S), UCC(S), UCA(S), UCG(S), GCU(A), GCC(A), GCA(A), GCG(A)	16	L,V,S,A	4
Q	mnm5s2UUG,CUG	CUA(L), CUG(L), CCU(P), CCA(P), CCG(P), CAU(H), CAC(H), GAA(E), CGA(R), CGG(R)	10	L,P,H,E,R	5
R	ICG,CCG,mnm5UCU,CCU	CCU(P), UGC(C), GGG(G)	3	P,C,G	3
S	GGA,CGA,cmo5UGA,GCU	UUU(F), UUC(F), UUA(L), UUG(L), CUU(L), CUC(L), CUA(L), CUG(L), GUU(V), GUC(V), GUA(V), GUG(V), CCU(P), CCC(P), CCA(P), CCG(P), ACU(T), ACC(T), ACA(T), ACG(T), GCU(A), GCC(A), GCA(A), GCG(A), CAU(H), CAA(Q), CAG(Q), AAU(N) AAC(N), GAA(E), UGU(C), UGC(C), AGA(R), AGG(R), GGU(G), GGC(G)	36	F,L,V,P,T,A,H,Q,N,E,C,R,G	13
T	GGU,CGU,cmo5UGU	UUU(F), UUC(F), UUA(L), UUG(L), CUU(L), CUC(L), CUA(L), CUG(L), AUU(I), AUC(I), AUA(I), AUG(M), GUU(V), GUC(V), GUA(V), GUG(V), UCU(S), UCC(S), UCA(S), UCG(S), CCU(P), CCC(P), CCA(P), CCG(P), GCU(A), GCC(A), GCA(A), GCG(A), AAA(K), AAG(K), AGU(S), AGC(S)	32	F,L,I,M,V,S,P,A,K	9
V	GAC,cmo5UAC	UUC(F), UUA(L), UUG(L), CUU(L), CUC(L), CUA(L), CUG(L), AUU(I), AUC(I), AUA(I), CCU(P), CCC(P), CCA(P), CCG(P), ACU(T), ACC(T), ACG(T), GCU(A), GCC(A), GCA(A), GCG(A)	21	F,L,I,P,T,A	6
W	CmCA	UUU(F), UUC(F), UUG(L), CAU(H), CAC(H), UGC(C)	6	F,L,H,C	4
Y	QUA	UUU(F), UUC(F), CCU(P), CAU(H), CAC(H), UGU(C), UGC(C)	7	F,P,H,C	4

Supplementary Table 4. List of codon-anticodon combinations that induced misdecoding. Misincorporating amino acids and the corresponding anticodon of tRNA are shown at the left. Note that we have not identified which anticodon was involved in misdecoding if there are multiple isoacceptor tRNAs for a particular amino acid; Any of the isoacceptors possibly induced misdecoding. Misincorporated codons and the corresponding amino acids are shown at the right, where numbers of codons and amino acids are also indicated.

second base of codon			
**U**	**C**	**A**	**G**
UUA(Leu)-QUU(Asn) [1]	CCU(Pro)-ac^{4}CAU(Met) [3]	UAU(Tyr)-ac^{4}CAU(Met) [1]	CGA(Arg)-QUU(Asn) [4]
UUG(Leu)-QUU(Asn) [1]	CCC(Pro)-ac^{4}CAU(Met) [3]	UAC(Tyr)-ac^{4}CAU(Met) [1]	GGG(Gly)-QUG(His) [1]
CUA(Leu)-QUU(Asn) [1]	CCA(Pro)-ac^{4}CAU(Met) [3]	CAU(His)-ac^{4}CAU(Met) [1]	
CUA(Leu)-GCA(Cys) [1]	CCA(Pro)-QUU(Asn) [1]	CAU(His)-CmCA(Trp) [2]	
CUG(Leu)-QUU(Asn) [3]	CCG(Pro)-GAU or LAU(Ile) [1]	CAC(His)-ac^{4}CAU(Met) [1]	
CUG(Leu)-GCA(Cys) [2]	CCG(Pro)-QUU(Asn) [3]	CAC(His)-CmCA(Trp) [2]	
GUA(Val)-QUU(Asn) [2]	GCU(Ala)-ac^{4}CAU(Met) [1]	AAG(Lys)-GAA(Phe) [1]	
GUA(Val)-GCA(Cys) [4]	GCC(Ala)-ac^{4}CAU(Met) [1]	AAG(Lys)-GCA(Cys) [1]	
GUG(Val)-QUU(Asn) [2]	GCA(Ala)-ac^{4}CAU(Met) [1]		
GUG(Val)-GCA(Cys) [4]	GCA(Ala)-GCA(Cys) [1]		
	GCG(Ala)-GCA(Cys) [1]		

Supplementary Table 5. Codon-anticodon combinations that induced 3-mismatch misdecoding. The second base of codon is used for their classification into four groups, U, C, A and G (indicated at the top). Combinations bearing C or U at the second base of codon account for 68% (21/31). Numbers in parentheses indicate the intensity of peptide.
